# Noncovalent Interactions Steer the Formation of Polycyclic Aromatic Hydrocarbons

**DOI:** 10.1021/jacs.4c03395

**Published:** 2024-08-07

**Authors:** Daniël B. Rap, Johanna G. M. Schrauwen, Britta Redlich, Sandra Brünken

**Affiliations:** FELIX Laboratory, Institute for Molecules and Materials, Radboud University, Nijmegen 6525 ED, The Netherlands

## Abstract

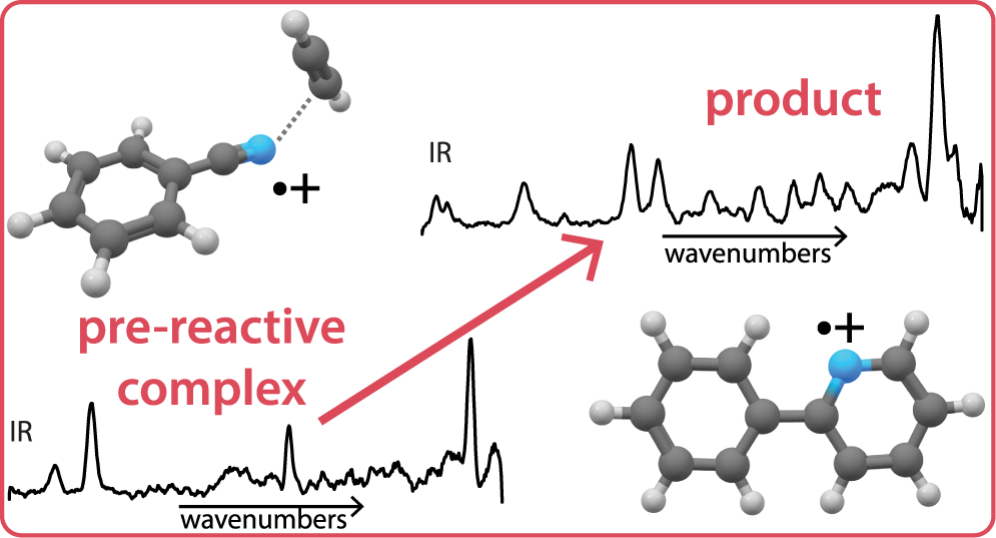

Aromatic molecules play an important role in the chemistry of astronomical environments such as the cold interstellar medium (ISM) and (exo)planetary atmospheres. The observed abundances of (polycyclic) aromatic hydrocarbons such as benzonitrile and cyanonaphthalenes are, however, highly underestimated by astrochemical models. This demonstrates the need for more experimentally verified reaction pathways. The low-temperature ion–molecule reaction of benzonitrile^•+^ with acetylene is studied here using a multifaceted approach involving kinetics and spectroscopic probing of the reaction products. A fast radiative association reaction via an in situ experimentally observed prereactive complex shows the importance of noncovalent interactions in steering the pathway during cold ion–molecule reactions. Product structures of subsequent reactions are unambiguously identified using infrared action spectroscopy and reveal the formation of nitrogen-containing, linked bicyclic structures such as phenylpyridine^•+^ and benzo-N-pentalene^+^ structures. The results, contradicting earlier assumptions on the product structure, demonstrate the importance of spectroscopic probing of reaction products and emphasize the possible formation of linked bicyclic molecules and benzo-N-pentalene^+^ structures in astronomical environments.

## Introduction

Noncovalent interactions play a pivotal role in many fields of chemistry. They govern folding mechanisms in proteins, the structure of DNA, molecular recognition^1,2^ in general, and are explored to steer catalytic processes.^3−5^ In gas-phase reactions the formation of a noncovalent prereactive complex in the entrance channel can have a dominant influence on the low-temperature behavior of their rate coefficients, leading to negative temperature behavior with reaction rates increasing as temperature decreases in cases where a positive reaction barrier exists,^6^ and even more complex temperature dependence in cases involving submerged barriers.^7,8^ Many earlier studies therefore aimed at the direct spectroscopic observation of prereactive complexes, using, e.g., microwave spectroscopy in a molecular beam,^9^ or infrared spectroscopy after their isolation in matrices^10^ or in helium nanodroplets.^11,12^

One realm were low-temperature bimolecular reactions play an important role is the interstellar medium (ISM), where numerous molecules have been detected, ranging from diatomics to larger organic molecules.^13^ One of the key questions in astrochemistry is how these organic molecules form under the often cold and rarefied conditions of the interstellar medium. Surprisingly, the presence of aromatic species remained elusive over decades.^14−17^ The first radio-astronomical detection of an aromatic molecule in the ISM happened only very recently, with the observation of benzonitrile (C_6_H_5_CN) in the cold molecular cloud TMC-1,^18^ which was followed rapidly by the detection of several other aromatic hydrocarbons and even PAHs,^19−22^ many of them containing a cyano (CN) group. The presence of some of these species is not unique for TMC-1 and, for example, benzonitrile has been observed in other star-forming regions.^23^

Signatures of large polycyclic aromatic molecules have not only been detected in the cold (∼10 K) and rarefied (number densities of the order 10^4^–10^5^ cm^–3^) interstellar medium, but also in warmer and denser regions, such as planetary/moon atmospheres.^24^ Using the mass spectrometer on board of the Cassini spacecraft, cationic species up to a mass-to-charge ratio (*m*/*z*) of 350 were detected revealing an immense complex chemistry taking place in the nitrogen and methane dominated atmosphere of Saturn’s moon Titan, more precisely in its ionosphere at altitudes of around 1000 km, where temperatures are between 100 and 200 K and neutral densities are of the order 10^10^ cm^–3^.^25−28^ Moreover, an infrared emission line from Titan’s upper atmosphere at 3.28 μm has been linked to the presence of large PAHs.^29,30^

The observation of PAHs in cold and dilute astronomical regions challenges the general view that astronomical PAHs are mainly formed through high-temperature combustion-like processes involving high reaction barriers, such as the hydrogen abstraction-acetylene addition (HACA) mechanism.^31,32^ The latter are likely to occur in circumstellar envelopes around late-type stars,^33−35^ however PAHs formed in these environments are unlikely to survive the journey through the ISM to regions where they were now detected.^36^ The lack of comprehensive information on formation routes of (polycyclic) aromatics is also evidenced by the observed high abundances of 1- and 2-cyanonaphthalene isomers in TMC-1 that are significantly underestimated by current astronomical models.^19^

In recent years, several studies investigated alternative molecular growth processes such as combustion-like pathways that are exoergic, fast, and barrierless and should proceed at the low temperatures of the ISM and in Titan’s atmosphere. These encompass neutral radical-mediated aromatization reactions, e.g., as calculated for the ethynyl addition mechanism (EAM)^37^ and experimentally studied in crossed-molecular beam setups, such as the ethynyl radical (C_2_H) and the cyano radical (CN) with 1,3 butadiene (C_4_H_6_) reactions forming benzene (C_6_H_6_)^38^ and pyridine (C_5_H_5_N),^39^ the formation of indene (C_9_H_8_)^40^ from methylidyne (CH) and styrene (C_6_H_5_C_2_H_3_) through a methylidyne addition-cyclization-aromatization (MACA) mechanism, or formation routes to anthracene and phenanthrene (C_14_H_10_) from naphthyl radicals (C_10_H_7_)^41^ and vinylacetylene (C_4_H_4_),^41^ often showing unexpected reaction pathways. An approach using the CRESU (Cinétique de Réaction en Ecoulement Supersonique Uniforme) method showed that formation of benzonitrile in a reaction of the cyano radical with benzene is fast and barrierless.^42^

Another class of important gas-phase reactions relevant for low-temperature PAH chemistry is ion–molecule reactions. To explain the abundance of heavy ionic species, reactions of ions with small and abundant hydrocarbon building blocks such as acetylene,^43^ ethylene and hydrogen cyanide are proposed.^44^ These ion–molecule reactions are often barrier-less and exothermic and can thus be relevant for colder astronomical environments. Many ion–molecule reactions of small ions with small neutral hydrocarbons have been experimentally investigated at room-temperature using ion cyclotron resonance (ICR) mass spectrometers and shown to be efficient.^45^ Different experimental approaches utilizing mass spectrometry, ion mobility and kinetics have been used to study ion–molecule reactions at room-temperature of astronomically relevant molecules such as pyridine, aniline and benzonitrile cations with small hydrocarbons^8,46,47^ or reactions between two cyclic building blocks such as benzene, pyridine, pyrimidine and naphthalene cations.^48−50^

However, there is still a lack of laboratory data to confirm the reaction pathways and rate coefficients used in the models at lower temperatures, and, even more importantly, to structurally characterize the potential reaction intermediates and products. For example, the ion–molecule reaction of the benzonitrile cation and acetylene presented here has been investigated earlier at room temperature by Soliman et al.^46^ using an ion mobility tandem mass spectrometer. In that study, two adducts with *m*/*z* 129 and *m*/*z* 155 are observed up to temperatures of 593 K and an eight-membered ring structure has been proposed for the product with *m*/*z* 155 based on quantum-chemical calculations. To assess the relevance of the reaction in astronomical environments such as Titan’s atmosphere, a kinetic investigation at lower temperatures (e.g., 150 K) and lower pressures is required. Moreover, since mass spectrometry alone is often insufficient to correctly infer the molecular structure of the products and possible branching ratios, spectroscopic methods are vital to unambiguously assign the structure of the reaction products and thereby verifying the hypothesized structure of *m*/*z* 155. Our group has recently used a combination of kinetic and infrared spectroscopic measurements to study the ion–molecule reaction between pyridine^•+^ (C_5_H_5_N^•+^) and acetylene (C_2_H_2_), previously studied using ion mobility mass spectrometry by Soliman et al.,^47^ and revealed an efficient formation of the endoskeletal nitrogen-containing-PAH (N-PAH) quinolizinium (C_9_H_8_N^+^) at low temperatures.^51^

**Figure 1:**
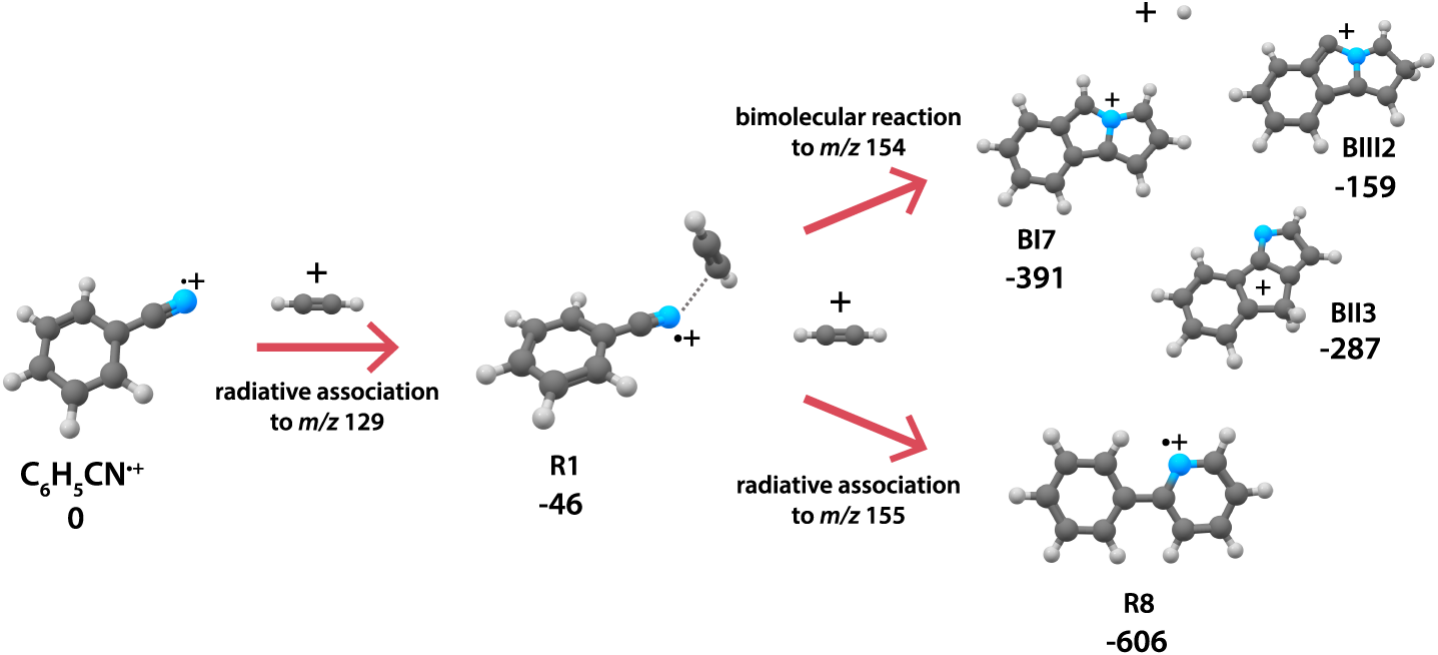
Schematic overview of the overall reaction of benzonitrile^•+^ (C_6_H_5_CN^•+^) with acetylene (C_2_H_2_) as spectroscopically determined in this work. Structures of the assigned intermediate **R1** (noncovalent acetylene-benzonitrile^•+^ complex), bimolecular products **BI7**, **BII3**, and **BIII2** (benzo-N-pentalene^+^ isomers) and radiative association product **R8** (2-phenylpyridine^•+^) are shown with energies (in kJ/mol) that correspond to the PES calculations from Figures 4 and 5.

Here, we report on a mass-spectrometric, infrared action spectroscopic, and computational approach to explore the mass-growth reactions from the isomer-selected benzonitrile radical cation, benzonitrile^•+^ (C_6_H_5_CN^•+^), with acetylene (C_2_H_2_) at low temperature (150 K) and pressures in a cryogenic 22-pole ion trap tandem mass-spectrometer.^52^ Kinetic reaction rate coefficient measurements revealed an efficient radiative association process for the first addition of acetylene to C_6_H_5_CN^•+^ and subsequent competing bimolecular (accompanied by H-loss) and radiative association steps. Isomeric structures of the reactant, intermediate and final products were elucidated in situ using rare-gas tagging and multiple-photon dissociation infrared action spectroscopy with the widely tunable and powerful infrared free-electron lasers at the FELIX Laboratory.^53^ The experimental broadband vibrational spectra were compared to electronic structure and vibrational frequency calculations. The in situ spectroscopic probing revealed that the first radiative association reaction step proceeds via a noncovalently bound acetylene-benzonitrile^•+^ complex (**R1**), followed by the formation of polycyclic molecules upon the addition of a second acetylene, with either multiple fused rings via a bimolecular H-loss reaction to benzo-N-pentalene^+^ (**BI7**) and its isomers benzo-N-pentaleneCH_2_^+^ (**BII3/BIII2**), or via an association reaction to a species with two covalently linked rings, 2-phenylpyridine^•+^ (**R8**) (see overview schematic in Figure 1). The reaction pathways were verified to be exothermic and barrier-less by electronic structure calculations of the potential energy surface, which represents evidence that large aromatic molecules can be formed efficiently via radiative association processes under interstellar medium and planetary atmosphere conditions. The isolation and spectroscopic characterization of the stabilized, noncovalently bound prereactive intermediate complex highlights the so-far widely neglected role of noncovalent interactions in the cold chemistry of ions with neutral molecules.

## Experimental and Theoretical Methods

### Kinetic Measurements

All experiments were performed with the FELion cryogenic ion trap apparatus^52,54^ stationed at the free-electron laser facility FELIX.^53^ More detail on the used methodologies is described in Rap et al.^51^ A liquid sample of benzonitrile (99.9% for HPLC, Sigma-Aldrich) was evaporated at room temperature and the vapor was ionized by the impact of 17 or 30 eV electrons. The benzonitrile ions were mass selected by a first quadrupole mass spectrometer and guided into the 22-pole ion trap that is mounted on a helium cryostat and was maintained here at around 150 K. A short (5–30 ms) and intense helium buffer gas pulse was introduced into the ion trap using a piezo valve to kinetically and internally cool the ions to close to the nominal trap temperature. A mixture of acetylene:helium (mixing ratios of 3:7 and 3:17 of C_2_H_2_:He were used) was continuously let into the trap through a leakage valve to proceed the ion–molecule reaction for specified trapping times ranging between 0 and 2600 ms. Typical number densities of acetylene between 6 × 10^9^ cm^–3^ up to 9 × 10^11^ cm^–3^ were used, associated with single collision conditions and collision times in the order of ∼1–100 ms. The reaction intermediates and products were extracted from the ion trap and detected by a second quadrupole mass spectrometer and a single ion detector. The number density of the neutral gas was determined by measuring the pressure of a hot-ionization gauge calibrated to a spinning-rotor gauge (MKS SRG3-EL). The kinetic curves were fitted using a set of ordinary differential equations (see Supporting Information) and the second-order reaction rate coefficients were determined using the obtained number density (Supporting Figure 4).

### Spectroscopic Measurements

The reaction intermediates and products formed were structurally identified with infrared multiple-photon dissociation (IRMPD) spectroscopy in situ in the cryogenic ion trap using the free-electron laser FEL-2 at the FELIX Laboratory.^53^ To ensure formation of the products before the arrival of the first FEL macro pulse, the mass selected benzonitrile^•+^ was reacted with a short (5–11 ms) and intense (total number densities of the order 10^15^ cm^–3^) pulse of acetylene–helium (the mixture was highly diluted with helium to prevent further reactions to obtain sufficient signal for *m*/*z* 129). We would like to note here that under these conditions termolecular processes dominate, leading to efficient collisional stabilization of association products. After pumping out the gas from the pulse, the ions were irradiated with intense (up to 30 mJ per pulse) and tunable infrared radiation provided by FEL-2 operating at 10 Hz in the 550–1700 cm^–1^ range with a FWHM of 0.5% of the center frequency. The amount of fragmentation/depletion upon resonant vibrational excitation of the ions was measured as a function of wavenumber to yield an infrared spectrum. A typical number of 26 macropulses were used to get sufficient fragmentation of the ions. The wavenumber was calibrated using an infrared spectrum analyzer with an accuracy of 1–2 cm^–1^ and the ion signal was normalized to the laser pulse energy (*E*), and number of laser pulses (*N*) to determine the relative cross section (*I*) according to
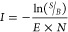
1with *S* the observed ion counts and *B* the baseline ion count number. The infrared spectrum of the reactant benzonitrile^•+^ has been measured using infrared predissociation spectroscopy (IRPD) using Ne-tagging with FEL-2 operating in the 550–1800 cm^–1^ range and using the third harmonic to reach the 2000–2400 cm^–1^ range, as detailed in Rap et al.^55^ Saturation depletion measurements^56^ were performed to determine the isomeric abundance of benzonitrile^•+^. Using a resonant wavelength, multiple laser pulses were used to fully deplete this isomer. The analysis of the depletion as a function of the number of laser pulses yielded the abundance of the canonical benzonitrile^•+^ (see Supporting Figures 2 and 3).

### Quantum Chemical Calculations

Molecular structures reported earlier in the literature^8,46^ as well as other plausible structures were investigated using density functional theory (DFT) calculations with Gaussian 16.^57^ The molecular geometries were optimized to their lowest energy using the B3LYP-GD3/N07D^58−61^ level of theory. The harmonic infrared spectra were determined and the frequencies were scaled with a typical scaling factor of 0.976 to account for anharmonic effects.^62^ The assigned molecules were further optimized and their infrared frequencies were determined at the B3LYP-GD3/6-311++G(2d,p) level of theory. The anharmonic infrared spectrum of the different benzo-N-pentalene^+^ isomers were calculated at the B3LYP-GD3/N07D level of theory using the VPT2 functionality of Gaussian to allow a better comparison with the experimental spectrum. To construct the potential energy surfaces, transition states were calculated at the B3LYP-GD3/N07D level of theory and evaluated using intrinsic reaction-coordinate calculations. All electronic energies were corrected for the zero-point vibrational energy. The noncovalent interactions have been visualized using NCI analysis^63^ with MultiWfn^64^ using the reduced electron density gradient:
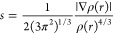
2with ρ the electron density. An isosurface (0.6) of the reduced density gradient (*s*) has been taken and colored using the values of s*ign*(λ_2_)ρ, with λ_2_ the second largest eigenvalue of the Hessian of the electron density, ranging from −0.03 to 0.02 (a.u.).

## Results and Discussion

### Spectroscopic Fingerprint of the Reactant Benzonitrile^•+^

The neutral benzonitrile molecule has been detected in the cold molecular cloud TMC-1^18^ and in other astronomical sources that are in the earlier stages of star-formation.^23^ The cationic form of benzonitrile has not been detected in the ISM yet, but it can be expected to exist in molecular clouds, photodissociation regions or Titan’s atmosphere due to UV and/or cosmic ray ionization processes. Earlier infrared spectroscopic data on the radical cationic,^55,65,66^ protonated,^67^ and electronically excited cationic^68^ form exists. Prior to the performed ion–molecule chemistry, we have spectroscopically characterized benzonitrile^•+^ using infrared predissociation (IRPD) spectroscopy of neon tagged ions using the free-electron laser FEL-2 at the FELIX Laboratory,^53^ see the Experimental and Theoretical Methods section and Rap et al.^55^ The infrared fingerprint spectrum in the 550–1800 cm^–1^ and the 2000–2400 cm^–1^ range is shown in Supporting Figure 1. A comparison with anharmonic frequency calculations is performed and the experimental infrared frequencies together with the assigned calculated vibrational modes are summarized in Supporting Table 1, showing an excellent match allowing for a clear assignment to the benzonitrile cation. The characteristic C≡N stretching mode of the benzonitrile cation lies ∼100 cm^–1^ lower compared to the neutral form.^69^ Saturation depletion measurements on vibrational bands that belong to the canonical benzonitrile cation structure show depletion values of >98(±5)% (Supporting Figures 2 and 3). Both the structural determination and the accompanying depletion scans enabled us to infer that the ionic reactant of the studied reaction is purely the canonical benzonitrile^•+^ (C_6_H_5_CN^•+^) structure and that no reactive distonic isomers^70^ are present.

### Rate Coefficient Measurements of the Benzonitrile^•+^ + C_2_H_2_ Reaction

**Figure 2:**
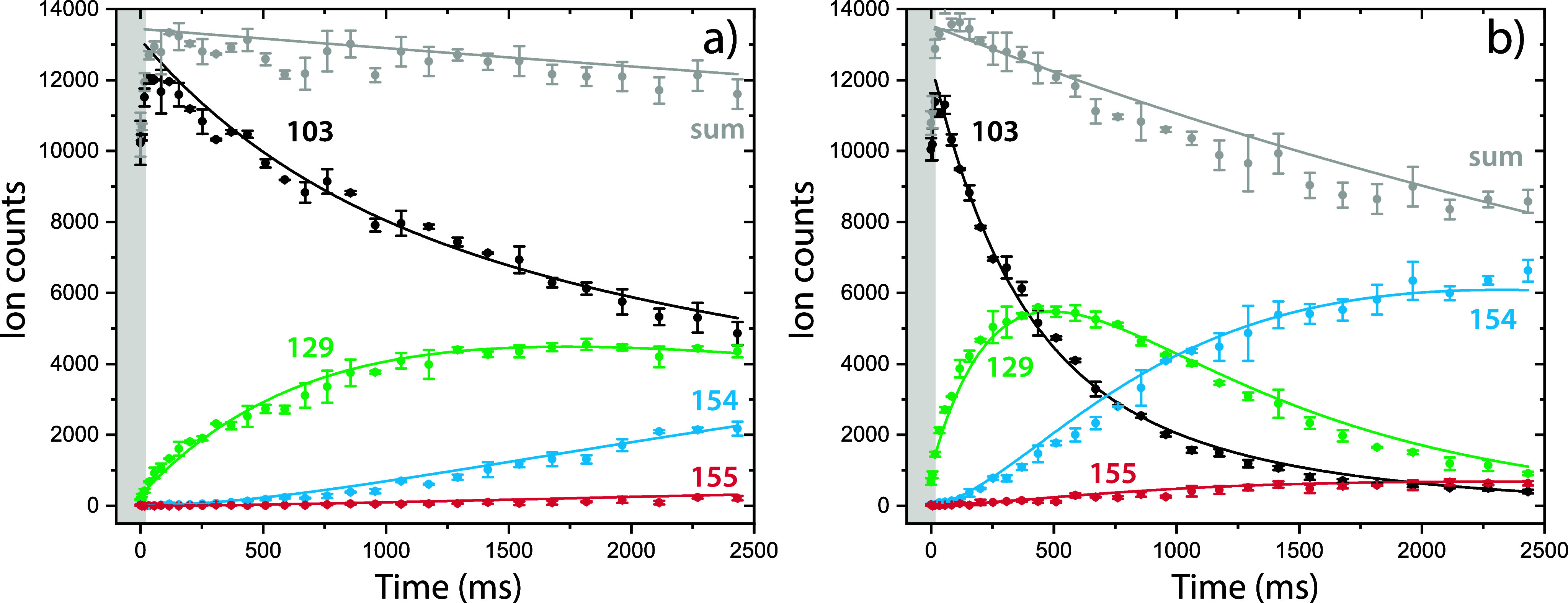
Exemplary kinetic plots of the ion–molecule reaction between benzonitrile^•+^ (C_6_H_5_CN^•+^) and acetylene (C_2_H_2_) performed at 150 K for a) low (1.23(±0.14) × 10^10^ cm^–3^) and b) high (4.3(±0.5) × 10^10^ cm^–3^) acetylene number density. The experimental ion counts of the reactant benzonitrile^•+^ (black), intermediate *m*/*z* 129 (green) and product structures with *m*/*z* 154 (blue) and *m*/*z* 155 (red) at different trapping times are plotted with dots and error bars. The plots are fitted with an ODE master equation containing the different reaction steps and plotted with a line. The gray dots indicate the sum of all ions and the decay has been implemented into the ODE model to account for the overall ion loss from the trap. The gray box indicates three-body collision conditions due to the He pulse used for trapping the ions in the beginning, these data points were not used in the fitting process.

To perform the ion–molecule reaction between C_6_H_5_CN^•+^ and C_2_H_2_, mass selected benzonitrile^•+^ (*m*/*z* 103) was stored and kinetically and internally cooled in a cryogenic 22-pole ion trap^52^ operating at a temperature of 150 K. Acetylene gas (*m* = 26 u) was led into the trap to initiate the reaction, and mass-growth processes toward multiple new molecules with *m*/*z* 129, 154, and 155 were observed. The formation was followed over time by adjusting the residence time of the ions in the trap. This procedure has been performed with various accurately determined acetylene number densities (Supporting Figure 4) and two exemplary kinetic profiles are shown in Figure 2.

From the characteristics of these plots, we can deduce information on the role of the different species in the whole reaction network. At conditions with a higher acetylene number density, we can see both the in- and decrease of ions with *m*/*z* 129 over time, indicating that this ion is the reaction intermediate upon the addition of the first acetylene to benzonitrile^•+^. Also, two product masses are observed at *m*/*z* 154, upon the addition of acetylene and loss of a hydrogen atom, and *m*/*z* 155, upon the direct addition of acetylene, to the intermediate *m*/*z* 129. Using an ordinary differential equation (ODE) model that encompasses all reactions in this system at varying acetylene number density, we were able to derive reaction rate coefficients (Table 1) of the individual reaction steps (more detail is given in the Supporting Information).

**Table 1:** Experimentally Determined Reaction Rate Coefficients of the Ion–Molecule Reaction between Benzonitrile^•+^ (C_6_H_5_CN^•+^) and Acetylene (C_2_H_2_)

reaction	rate coefficient (cm^3^s^–1^)	type	label	number density range C_2_H_2_ (cm^–3^)
103^·+^ → 129^·+^	3.8(±0.4) × 10^–11^	radiative association	*k*_RA_	6.1 × 10^9^ – 2.6 × 10^11^
129^·+^ → 154^+^	1.3(±0.3) × 10^–11^	bimolecular	*k*_bi_	6.1 × 10^9^ – 2.6 × 10^11^
129^·+^ → 155^·+^	3.6(±0.7) × 10^–12^	radiative association	*k*_RA,C11_	1.2 × 10^10^ – 8.7 × 10^10^
129^·+^ → 103^·+^	5.6(±0.9) × 10^–12^	collision-induced dissociation	*k*_CID_	4.1 × 10^10^ – 8.7 × 10^11^

The formation of the reaction intermediate with *m*/*z* 129 proceeds via a fast effective bimolecular association process with a reaction rate coefficient *k*_EFF-BI_ of 6.2(±0.3) × 10^–11^ cm^3^ s^–1^. A small dependence on the acetylene (or acetylene:He) number density was observed indicating a contribution by termolecular stabilization (Supporting Figure 5). The radiative association rate coefficient (*k*_RA_), where the product is stabilized only by the emission of a photon, is thus determined at a slightly lower value of 3.8(±0.4) × 10^–11^ cm^3^ s^–1^, and is reported in Table 1. Similar relatively high radiative association rate coefficients have been observed for the reaction of other larger aromatic molecules such as benzonitrile and NO^+^ (2.8 × 10^–11^ cm^3^ s^–1^)^71^ and pyridine^•+^ and acetylene (8.0(±3.5) × 10^–11^ cm^3^ s^–1^).^51^ A similar reaction rate constant of 4.2(±2.5) × 10^–11^ cm^3^ s^–1^ was previously measured for the ion–molecule reaction between benzonitrile^•+^ and acetylene using ion-mobility tandem mass spectrometry at 304 K, which likely includes contributions from termolecular stabilization due to the higher pressures used in that experiment.^46^ The radiative association rate coefficient obtained here is larger than the effective bimolecular rate coefficients obtained by Shiels et al. for the related distonic benzonitrile radical ions, and is thus in line with their observed trend of increasing reactivity with increasing relative barrier energy and EA-IP curve crossing relationship.^8^ It is, however, significantly lower than what one would expect from their model. We attribute this to the fact that here we report the radiative association rate coefficient, whereas in their ion trap study much higher pressures were used, leading likely to the measurement of saturated termolecular rate coefficients, which can be orders of magnitude larger, as we have demonstrated earlier for the reaction of pyridine^•+^ with acetylene.^51^ The branched sequential reaction toward *m*/*z* 154 and *m*/*z* 155 proceeds via a bimolecular reaction with a lower rate coefficient *k*_bi_ of 1.3(±0.3) × 10^–11^ cm^3^ s^–1^ and another radiative association process with a rate coefficient *k*_RA,C11_ of 3.6(±0.7) × 10^–12^ cm^3^ s^–1^, respectively. Also, we included a backward reaction from the intermediate *m*/*z* 129 toward the precursor *m*/*z* 103, that significantly improved the ODE model. This so-called collision-induced dissociation (CID) reaction may indicate the presence of a weakly bound system in the reaction pathway that can be fragmented upon collision by another molecule. The ratio between the forward (*k*_EFF-BI_) and backward (*k*_CID_) reaction rate coefficient is determined to be 11.2(±1.9) indicating that the intermediate *m*/*z* 129 is overall efficiently stabilized.

### Structural Elucidation of the Products by Spectroscopy

**Figure 3:**
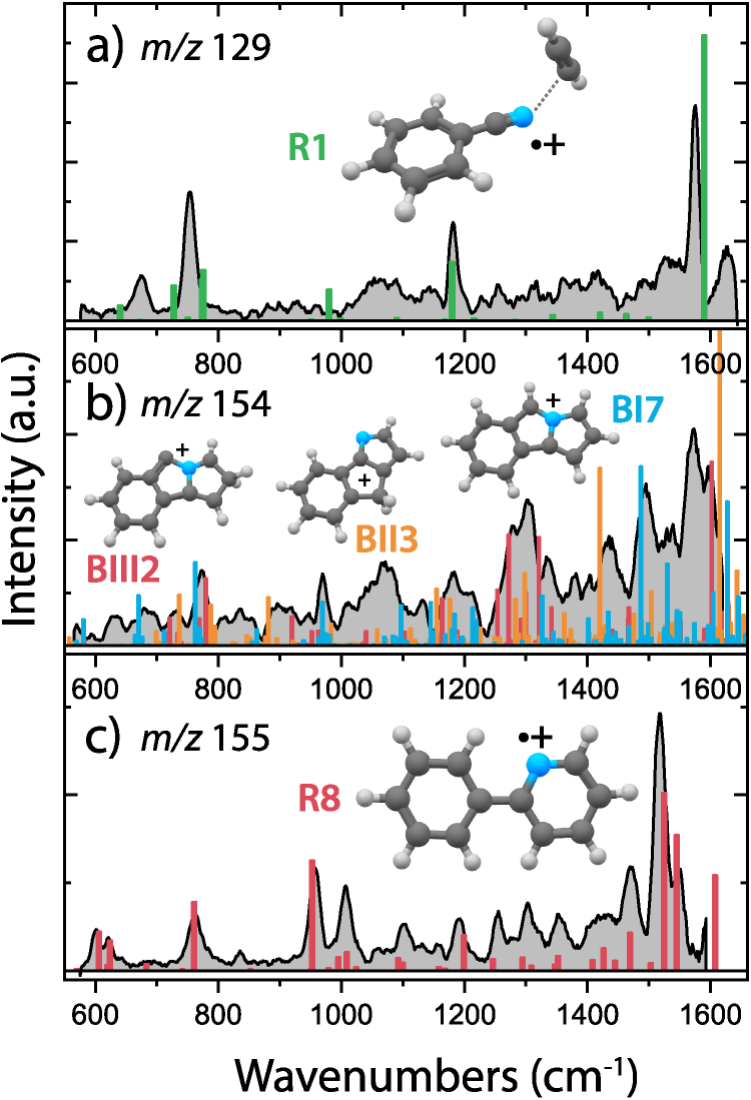
Experimental infrared spectra (gray) of the (a) *m*/*z* 129, (b) *m*/*z* 154, and (c) *m*/*z* 155 reaction intermediates and products of the ion–molecule reaction between benzonitrile^•+^ (C_6_H_5_CN^•+^) and acetylene (C_2_H_2_). Calculated infrared frequencies of the assigned structures are shown as sticks for (a) the noncovalent acetylene-benzonitrile^•+^ complex (**R1**, green), (b) benzo-N-pentalene^+^ (**BI7**, blue) and benzo-N-pentaleneCH_2_^+^ isomers (**BII3**, orange/**BIII2**, red) and (c) 2-phenylpyridine^•+^ (**R8**, red). The theoretical spectra of the *m*/*z* 129 and *m*/*z* 155 ions are calculated at the harmonic B3LYP-GD3/6-311++G(2d,p) level of theory, whereas the spectra of the *m*/*z* 154 isomers have been calculated at the anharmonic B3LYP-GD3/N07D level of theory.

Based solely on mass spectrometry and kinetic measurements one cannot elucidate the chemical structure of the reaction products and determine if covalent bonds have been formed. In this study, we add infrared action spectroscopy as a versatile experimental tool to investigate the structures of the ions formed in the reaction. Using the free-electron laser FEL-2 at the FELIX Laboratory,^53^ we measured the infrared spectra of the intermediates and reaction products in the 550–1700 cm^–1^ wavenumber range using infrared multiple-photon dissociation (IRMPD) spectroscopy. A comparison between the experimental spectrum and calculated vibrational bands of the assigned structure(s) is shown in Figure 3.

The intermediate *m*/*z* 129 species can be assigned to a noncovalent complex of benzonitrile^•+^ and acetylene (**R1**) based on the good overlap between experimental and calculated vibrational modes (Figure 3a). Also, the observed facile dissociation behavior upon vibrational excitation (>95% depletion on the 754 cm^–1^ band, compared to ∼20–40% for the covalently bound species with *m*/*z* 155 using similar laser energy and number of pulses) points toward a weakly bound species. The assignment to the noncovalent acetylene-benzonitrile^•+^ complex with a binding energy of 46 kJ/mol does agree with the incorporated CID process in the ODE model. Another isomer with *m*/*z* 129 such as the N-PAH isoquinoline^•+^ can be excluded based on the discrepancy between the experimental spectrum and calculated vibrational modes of these isomers (Supporting Figure 6). The feature at 674 cm^–1^ may be explained by a minority of covalently bound N-acetylene-benzonitrile^•+^ (**R4**) (Supporting Figure 6). Only a low abundance is expected based on the signal intensities at 674 and 1180 cm^–1^, and the nonobserved predicted 1270 cm^–1^ band, and, therefore, the intensity of the major experimental features can only be explained by the noncovalent acetylene-benzonitrile^•+^ complex as the dominant isomer. No infrared signatures of other covalently bound isomers **R2** and **R3** on the PES are observed above the noise level, see Supporting Figure 6.

**Figure 4:**
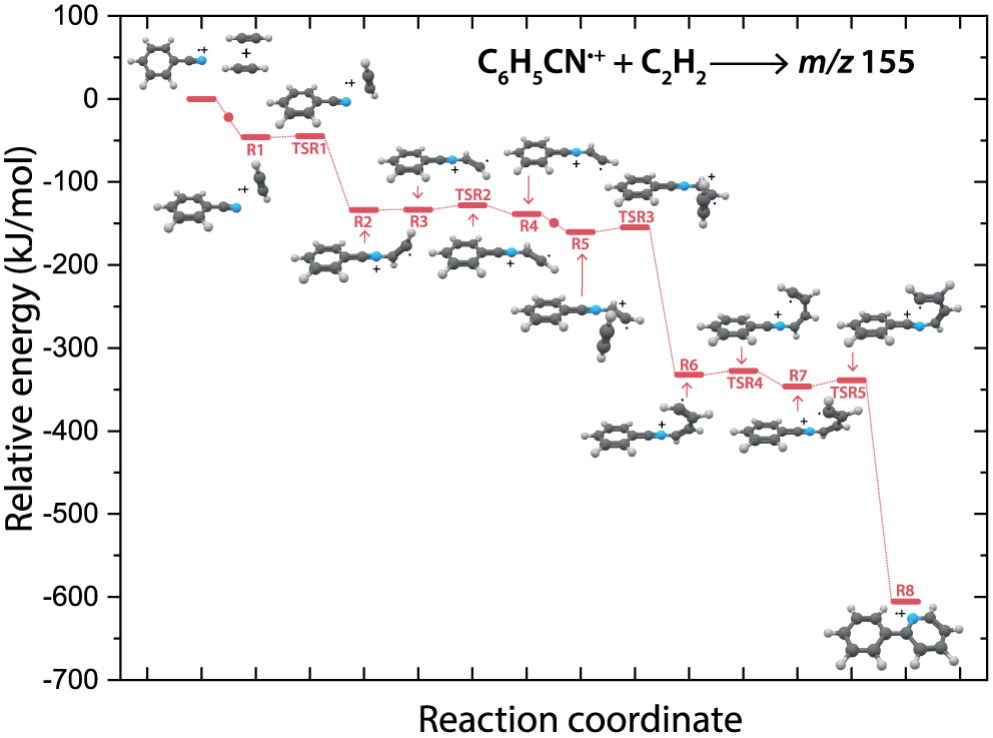
PES of the benzonitrile^•+^ (C_6_H_5_CN^•+^) with acetylene (C_2_H_2_) reaction toward the observed 2-phenylpyridine^•+^ (**R8**, *m*/*z* 155, red pathway). The small red dots along the pathway display the addition of a new acetylene molecule. The energies are calculated at the B3LYP-GD3/N07D level of theory and are corrected for the zero-point vibrational energy (values are provided in Supporting Table 2).

We would like to note here that for the spectroscopic probing experiment much higher overall neutral pressures were used than for the kinetic studies, see the Experimental and Theoretical Methods section. Under these high-pressure conditions, termolecular collisions can readily stabilize the noncovalent reaction complex. It is likely that, under single-collision conditions as used for the kinetic measurements, more of the other species that exist along the reaction coordinate (Figure 4), such as the covalently bound structures **R2**/**R3**/**R4** are formed as well. These covalently bound structures have been assigned by Soliman et al.^46^ based on their stability when formed at a higher reaction temperature of 593 K. At this temperature, sufficient energy is available to cross the forward barrier (**TSR1**, calculated height ∼1 kJ/mol, but likely attributed with large error margins due to the chosen level of theory) to covalently bound intermediates. The spectroscopic characterization of the noncovalent complex here establishes that the formation of such complexes can play a role in the first steps of ion–molecule reactions at lower temperatures.

**Figure 5:**
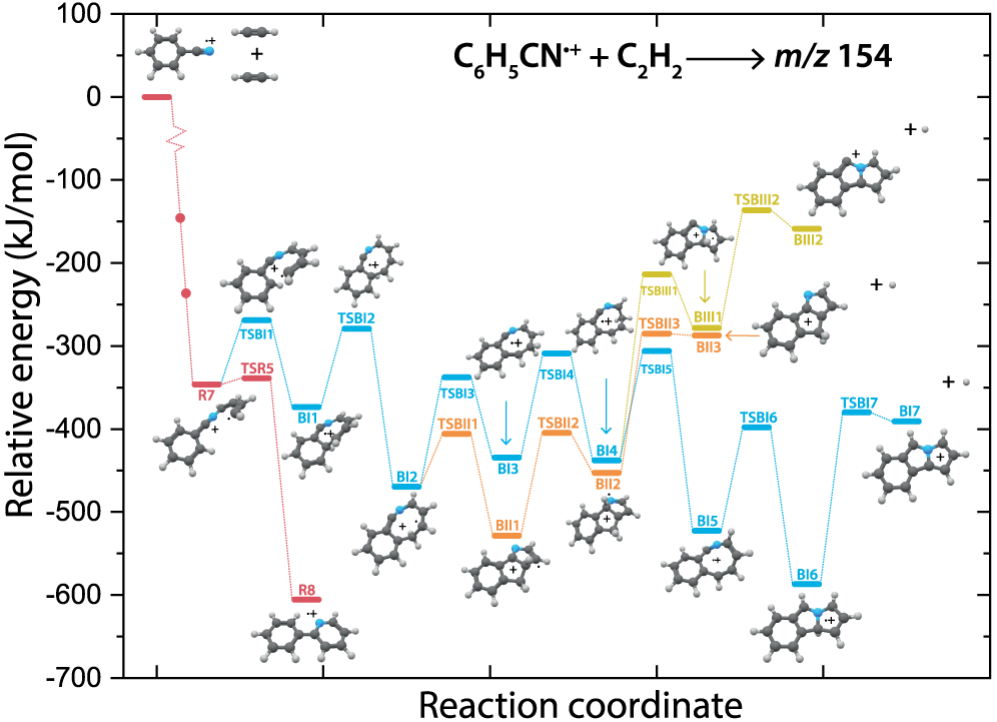
PES of the benzonitrile^•+^ (C_6_H_5_CN^•+^) with acetylene (C_2_H_2_) reaction toward the *m*/*z* 154 isomers benzo-N-pentalene^+^ (**BI7**, blue pathway), benzo-N-pentaleneCH_2_^+^ (**BII3**, orange pathway) and benzo-N-pentaleneCH_2_^+^ isomer (**BIII2**, yellow pathway). The first part of the reaction pathway (red) up to **R7** follows the calculated path from Figure 4. The small dots along the pathway display the addition of a new acetylene molecule. The energies are calculated at the B3LYP-GD3/N07D level of theory and are corrected for the zero-point vibrational energy (values are provided in Supporting Table 3).

For both products with *m*/*z* 154 and 155, we see the formation of structurally quite different, polycyclic molecules, with either multiple fused rings in the case of benzo-N-pentalene^+^ species (**BI7**/**BII3**/**BIII2**, Figure 3b) or covalently linked rings for 2-phenylpyridine^•+^ (**R8**, Figure 3c), respectively. A good agreement between the calculated and experimental spectrum of 2-phenylpyridine^•+^ points to the formation of a single isomer in the *m*/*z* 155 product channel. The broadening of the experimental features in the *m*/*z* 154 product spectrum indicates the presence of multiple isomers. A comparison with calculated anharmonic infrared spectra of three benzo-N-pentalene^+^ isomers shows remarkable similarity with the experimental spectrum for all three isomers (Supporting Figure 8). Although most of the features can be explained by one isomer, the canonical benzo-N-pentalene^+^ (**BI7**), additional contributions of the other isomers (**BII3** and **BIII2**) improve the spectral match as shown by a convoluted spectrum consisting of all three isomers (convoluted spectrum in Supporting Figure 9). The latter two isomers lie at higher energies relative to benzo-N-pentalene^+^ (Figure 5). Therefore, it is more likely that these are formed in lower abundance. Other product isomers such as protonated cyano-naphthalene^+^ (*m*/*z* 154) and protonated benzo-N-pentalene^•+^ (*m*/*z* 155) can be excluded based on the spectroscopic information (Supporting Figures 7 and 10).

A comparison with previous experiments by Soliman et al.^46^ that dominantly show the formation of *m*/*z* 155 confirms the hypothesized covalent nature of the product. However, a different structure than their proposed eight-membered ring structure (**BI5**) is spectroscopically probed here. We observe that the experimentally assigned lower energy structure (**R8,** 83 kJ/mol lower in energy) consists of two linked six-membered rings. Furthermore, in our experiments we predominantly see the product formation to *m*/*z* 154 which involves a bimolecular reaction with the loss of a hydrogen atom, in contrast to Soliman et al.^46^ where the product with *m*/*z* 154 was hardly observed. This is likely due to the significantly higher pressure (around 1 mbar) in the earlier study supporting three-body (termolecular) collision conditions, making the bimolecular H-loss channel toward *m*/*z* 154, where the reaction energy is released by the loss of a hydrogen atom, slower than the competing association reaction.

### Reaction Network Proposed by Spectroscopy, Kinetics, and Energetics

With the information obtained from the kinetic and spectroscopy measurements we can construct a molecular growth network of the benzonitrile^•+^ with acetylene ion–molecule reaction using quantum chemical calculations. By performing electronic structure calculations using density functional theory (DFT), we have calculated the potential energy surface (PES) for the formation of intermediate *m*/*z* 129 and the products *m*/*z* 154 and 155. The complete reaction pathway toward the product 2-phenylpyridine^•+^ (*m*/*z* 155) is shown in Figure 4.

Generally, the whole reaction is exothermic by 606 kJ/mol. The spectroscopically observed noncovalent intermediate (**R1**) can also be located on this PES. The structural rearrangement from this prereactive complex to a structure where the acetylene is attached to the nitrogen atom (**R5**) involves a small barrier of ∼1 kJ/mol (**TSR1**), that is overall submerged, and from this isomer a subsequent addition of acetylene forms a C_4_H_4_ chain (**R7**) that can close (**TSR5**) to form a second six-membered ring yielding 2-phenylpyridine^•+^ (**R8**).

The spectroscopically determined benzo-N-pentalene^+^ isomeric products (*m*/*z* 154) are structurally surprisingly different from 2-phenylpyridine^•+^ and exist on a competing PES as shown in Figure 5.

The first part of the pathway toward N–C_4_H_4_-benzonitrile^•+^ (**R7**) is the same as for the calculated reaction toward 2-phenylpyridine^•+^ (Figure 4). The transition state **TSBI1**, analogous to **TSR5**, displays the ring closure onto the existing six-membered ring and is calculated to form an eight-membered ring (**BI1**). Multiple branched pathways differing by the sequence of hydrogen migration and ring-closing processes are determined.

An earlier proposed structure for *m*/*z* 155 containing an eight-membered ring (**BI5**)^46^ lies on this potential energy surface. However, a structure containing multiple pentagonal rings (**BI6**) that is formed upon ring-shrinkage is calculated to be lower in energy. The final step involves the loss of a hydrogen atom to yield the benzo-N-pentalene (**BI7**) structure which is one of the spectroscopically observed isomers. The other less-abundant spectroscopically observed isomers benzo-N-pentaleneCH_2_^+^ (**BII3,** −287 kJ/mol) and benzo-N-pentaleneCH_2_^+^ isomers (**BIII2,** −159 kJ/mol), are higher in energy than benzo-N-pentalene^+^ (**BI7,** −391 kJ/mol), and are formed via higher lying transition states. Generally, all species are formed via the formation of an eight-membered ring, hydrogen migration processes and subsequent ring shrinkage to form two fused five-membered (pentagonal) rings which involve only submerged barriers with respect to the entrance energy. Interestingly, an analogous pure carbon benzo-pentalene^+^ structure has been experimentally observed in the fragmentation chemistry of the N-PAHs acridine and phenanthridine.^72^

Another pathway from the N-acetylene-benzonitrile^•+^ (**R4**) intermediate species involving a hydrogen migration from the ring toward the acetylene group requires the molecules to exist in an energetically unfavorable geometry (∠C–C–N = 151.6°) yielding an endothermic barrier of 5.9 kJ/mol (Supporting Figure 11). Therefore, the reaction on the acetylene side chain is more favorable leading to the diacetylene (−C_4_H_4_) group. This contrasts with previously determined reaction pathways of pyridine^51^ where hydrogen migration is found to be present.

**Figure 6:**
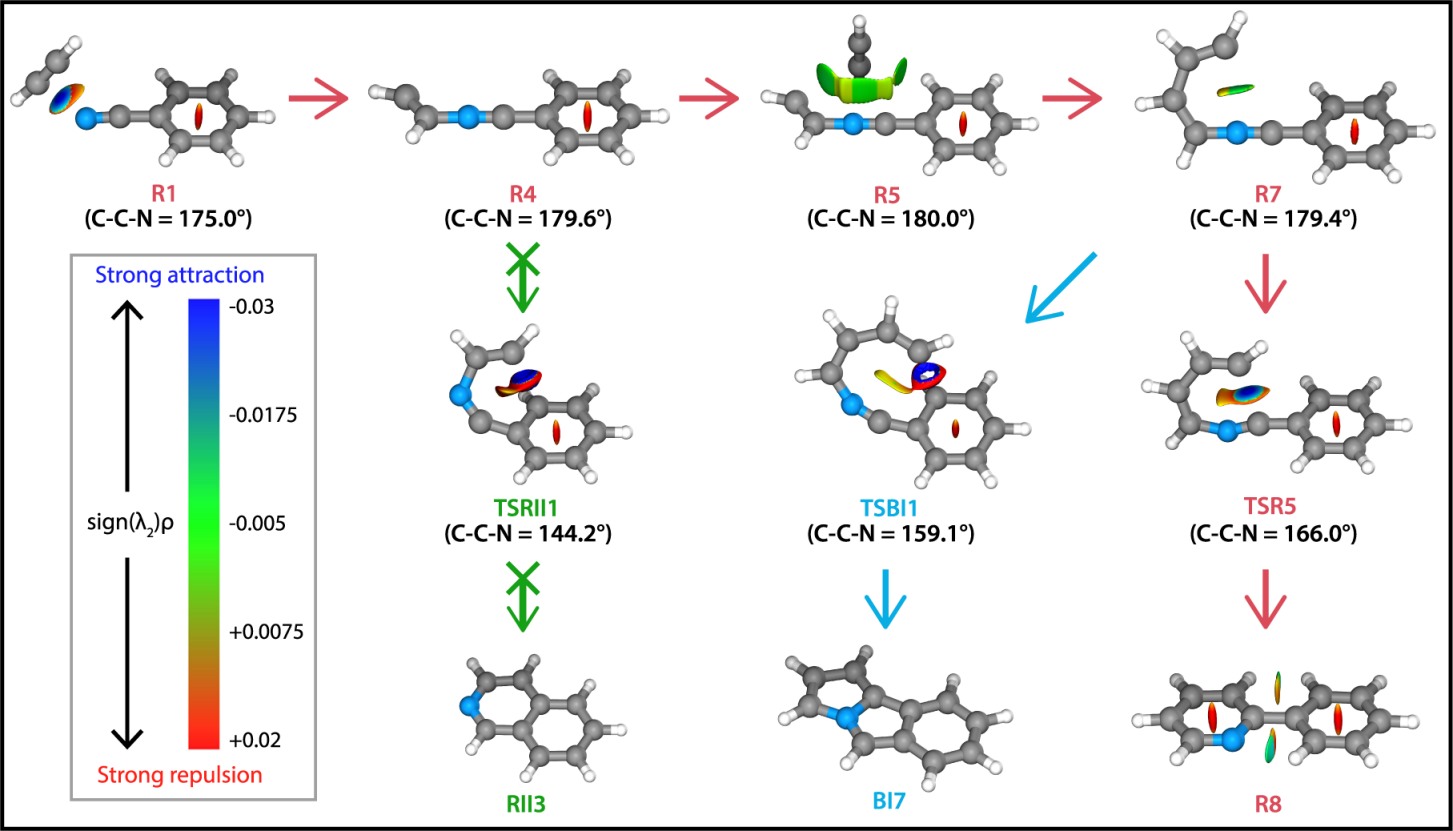
NCI plots of intermediate and transition states of the reaction pathways toward 2-phenylpyridine^•+^ (**R8**, red arrows), benzo-N-pentalene^+^ (**BI7**, blue arrows) and isoquinoline^•+^ (**RII3**, green arrows). The strengths of the NCIs are shown by the color spectrum ranging from red (strong repulsion), green (weak attraction) and blue (strong attraction). The calculated angle of the C–C–N group is shown next to the structures.

To understand the preference of the reaction toward 2-phenylpyridine^•+^ and benzo-N-pentalene^+^ we have visualized the noncovalent interactions (NCIs) that are involved in the reaction pathways (Figure 6). The NCIs have been calculated using the reduced electron density gradient approach^63^ that visualizes the location, strengths and nature of the different intra- and intermolecular interactions of the molecules (see the Experimental and Theoretical Methods section). This approach has previously been applied to investigate the NCIs of energy minima and transition states of other reactions.^73,74^

First of all, the reactions toward both products are favored by the formation of the two noncovalent acetylene complexes **R1** and **R5** upon the first and second acetylene addition, respectively. A strong attractive ion-induced dipole interaction (indicated by the blue disc) is shown for **R1**, whereas the other acetylene complex **R5** contains weak van der Waals (vdW) interactions indicated in green. Also, the N–C_4_H_4_-benzonitrile^•+^ (**R7**) structure is favored by a weak vdW interaction that retains a structure suitable as starting point for both **TSR5** and **TSBI1** to proceed toward ring-closure.

In contrast, the intermediate **R4** contains no preferential orientation caused by noncovalent interactions that benefits the ring-closure toward isoquinoline^•+^ after the first acetylene addition (**RII3**) (Supporting Figure 12). Moreover, the sp-hybridized atoms from the C–C–N group in **TSRII1** are significantly perturbed (calculated angle ∠C–C–N = 144.2°) from their energetically favorable orientation of ∼180°. This angle is also substantially lower than the equivalent angle in the transition states **TSBI1** (159.1°) and **TSR5** (166°) leading to the products benzo-N-pentalene^+^ and 2-phenylpyridine^•+^, respectively. This is further proven by the corresponding electronic energies of 10.8 kJ/mol for **TSRII1** compared to −269.0 kJ/mol and −338.7 kJ/mol, relative to the entrance channel, for benzo-N-pentalene^+^ and 2-phenylpyridine^•+^, respectively. The NCI calculations also show that 2-phenylpyridine^•+^ has a weak attractive interaction between the nitrogen atom and a neighboring CH group from the other aromatic ring causing the planarity of the system.

Altogether, the strong attractive interactions and weaker vdW interactions along the reaction pathway that both bind the acetylene reaction partner and lock a favorable conformation for further ring-closure to benzo-N-pentalene^+^ and 2-phenylpyridine^•+^, as well as the high bond angle strain of the transition state toward isoquinoline^•+^, explain the spectroscopically observed product structures.

### Astrochemical Implications

The astrochemical relevance of the chemical system studied here is demonstrated by multiple important facets:

First, the reactivity of aromatic species is significantly enhanced by the presence of nitrogen heteroatoms as observed here for benzonitrile^•+^ where the product structures have formed a new N–C bond, and earlier observed in other studies containing nitrogen-substituted aromatic molecules such as pyridine and pyrimidine.^47,51^ The reaction of the pure hydrocarbon and aromatic benzene^•+^ with acetylene^75^ and another analogous reaction between phenylacetylene^•+^ and acetylene^46^ show significantly lower reaction rate coefficients of 3.7(±0.8) × 10^–14^ cm^3^ s^–1^ (623 K) and 1.5(±1.1) × 10^–12^ cm^3^ s^–1^ (302 K), respectively, compared to what was measured here for benzonitrile^•+^ (*k*_RA_ = 3.8(±0.4) × 10^–11^ cm^3^ s^–1^).

Second, both the first reaction step via the noncovalent prereactive complex (**R1**) and the sequential reaction toward 2-phenylpyridine^•+^ proceed via fast radiative association reactions. This reaction type plays an important role^76^ in low-density regions of the ISM and Titan’s and other (exo)planetary atmospheres where two-body collision conditions are present, and radiative association becomes even more favorable for larger molecules due to their larger density of states. Radiative association processes point thus toward new potential reaction pathways for interstellar aromatic chemistry. The stabilization and spectroscopic detection of the noncovalent intermediate complex (**R1**), also denoted as prereactive complex, highlights the role of noncovalent interactions in the cold chemistry of ions with neutral molecules, as has been previously discussed for the reactions of distonic benzonitrile, pyridine and aniline cations with acetylene.^8,77^ Similarly, this has been discussed for radical-neutral bimolecular reactions of phenyl type radicals with neutral hydrocarbons which are dictated by attractive long-range interactions and the formation of noncovalent (or vdW) complexes.^36^ In addition, intramolecular attractive interactions, such as vdW forces observed in the N–C_4_H_4_-benzonitrile^•+^ (**R7**) structure display the importance of noncovalent interactions that may steer the reaction toward specific product structures. The formation of the cis-isomeric form of the C_4_H_4_-group within **R7** is favored by attractive vdW forces and thereby favors sequential ring-closure to 2-phenylpyridine^•+^.

Third, the structure of the reaction products includes polycyclic species that consist both of fused aromatic rings and those that are covalently linked through a single bond. Similar linked bicyclic molecules have been detected in ion–molecule reactions of benzyne^•+^ isomers and acetylene by Rap et al.^78^ and upon ion–molecule reactions of two cyclic building blocks.^48−50^ Both experimental detections stress the importance of studying the chemistry of these so-called linked (singly bonded) polycyclic aromatic compounds using both laboratory experiments and quantum chemical calculations. An important mechanism toward analogously linked molecules such as biphenyl and terphenyl proceeds via radical-neutral reactions following the phenyl addition cyclization (PAC) mechanism.^79−82^ This growth mechanism may have significant importance for interstellar chemistry as the growth occurs via the addition of whole benzene/phenyl^•^ moieties. In this study, we form an analogous molecule, 2-phenylpyridine^•+^, via subsequent addition of acetylene. The formed product can act as starting point for further growth processes similar to the PAC mechanism which has been demonstrated to be also efficient for cationic molecules.^83,84^

Altogether, the experiments performed at 150 K show that these reactions efficiently occur in cold conditions and can therefore be relevant for the chemistry in the cold ISM, as well as for the formation of large ions that have been observed in the ionosphere of Titan.^27,85^ In cold molecular clouds like TMC-1, ionization fractions caused by cosmic ray ionization are typically rather low,^86^ of the order 10^–7^, and in addition competing reactions of the benzonitrile cation and the reaction intermediates, e.g., with atomic and molecular hydrogen, need to be considered within a complex astrochemical model to evaluate the importance of the proposed reactions.

Titan’s ionosphere, on the other hand, has an overall high ion density^87^ of up to 10^3^ cm^–3^, acetylene was found by Cassini-Huygens to be the third most abundant hydrocarbon,^88^ and substantial abundances of benzonitrile have been predicted based on model calculations.^89^ The related pyridine cation, for example, has been detected mass-spectroscopically in Titan’s ionosphere at comparably high densities of around 10 cm^–3^.^87^ Additional pathways for direct formation of the benzonitrile cation via association reactions have been proposed.^90^ A closer look at the masses measured by the Cassini spacecraft^27^ reveal strong features around *m*/*z* 103, 129, and 155 which are attributed to molecular formulas similar as observed in the benzonitrile^•+^ and acetylene reaction. Moreover, singly bonded (linked) aromatic compounds (polyphenyls^91^ are expected to be the basis of the (co)polymeric structures that Titan’s tholins are expected to consist of.^28,91,92^ Further systematic experimental studies into the reaction pathways of related nitrogen-containing polycyclic hydrocarbons need to be conducted in order to discover new pathways and new molecular species, which ultimately act as candidates for infrared and radio-astronomical searches.

## Conclusions

By combining experimental kinetics and spectroscopic studies with quantum-chemical descriptions of the energetics and noncovalent interactions, we could obtain a comprehensive insight into ion–molecule chemistry on the example of the reaction of benzonitrile^•+^ with acetylene. The bimolecular reactions of benzonitrile^•+^ with acetylene show the formation of multiple compounds including linked six-membered rings and polycyclic species consisting of a pentalene structural motif. The spectroscopic detection of a noncovalent prereactive complex highlights the importance of noncovalent interactions in ion–molecule reactions relevant to the chemistry in cold regions of space.

## Supporting Information Available

Experimental infrared spectra, comparisons with calculated infrared spectra, saturation depletion scans, kinetic results, and potential energy surfaces (PDF)
