# Supplementary material for: Noncovalent Interactions Steer the Formation of Polycyclic Aromatic Hydrocarbons
Source: J Am Chem Soc. 2024 Aug 7;146(33):23022–33. doi: 10.1021/jacs.4c03395 (PMC11345775; doi:10.1021/jacs.4c03395)
Supplement: Supplementary file 1 — ja4c03395_si_001.pdf [file ja4c03395_si_001.pdf]

Supplementary Information:

Noncovalent Interactions Steer the Formation of Polycyclic Aromatic Hydrocarbons

Daniël B. Rap, Johanna G.M. Schrauwen, Britta Redlich and Sandra Brünken*

Radboud University, FELIX Laboratory, Institute for Molecules and Materials, Toernooiveld 7, 6525 ED Nijmegen, The Netherlands, *E-mail: sandra.bruenken@ru.nl

Infrared spectrum of benzonitrile$^{\bullet+}$

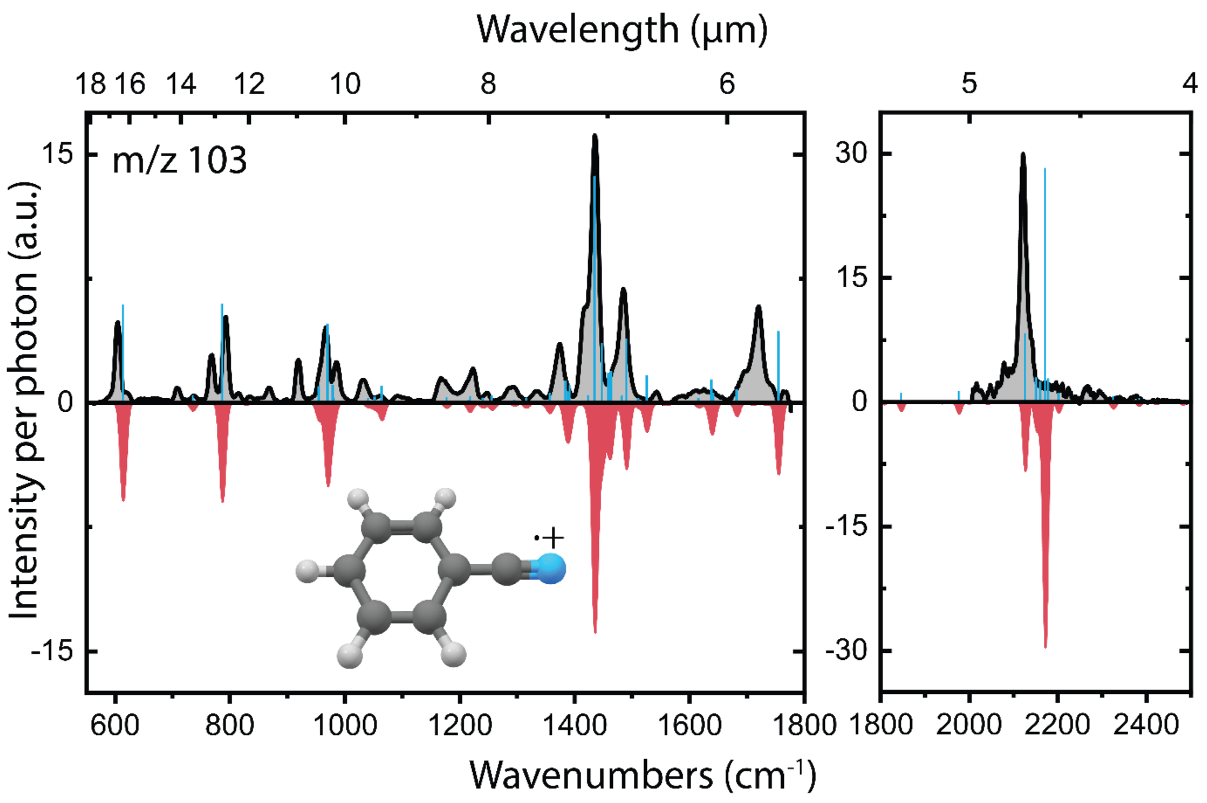

Supplementary Figure 1: Experimental infrared spectrum (grey) of the reactant benzonitrile$^{\bullet+}$ ($C_6H_5CN^{\bullet+}$) with *m/z* 103. The calculated anharmonic infrared frequencies are shown as blue sticks and are convoluted with a Gaussian line-shape with a 5 cm^{-1} width to account for (experimental) broadening effects. The latter is shown as the red spectrum.

Saturation depletion of benzonitrile$^{\bullet+}$

Details on the saturation depletion method are described in detail by Marimuthu et al.[1] but a brief explanation is given here. Multiple laser pulses, resonant with a vibrational mode of one isomer, are used to burn away the specific isomer from the trap (blue curves, **Supplementary Figure 2 and 3**). As a background measurement, the depletion due to laser heating effects at a non-resonant wavelength is similar determined (orange curves, **Supplementary Figure 2 and 3**). From both measurements, the relative depletion of this isomer can be determined.

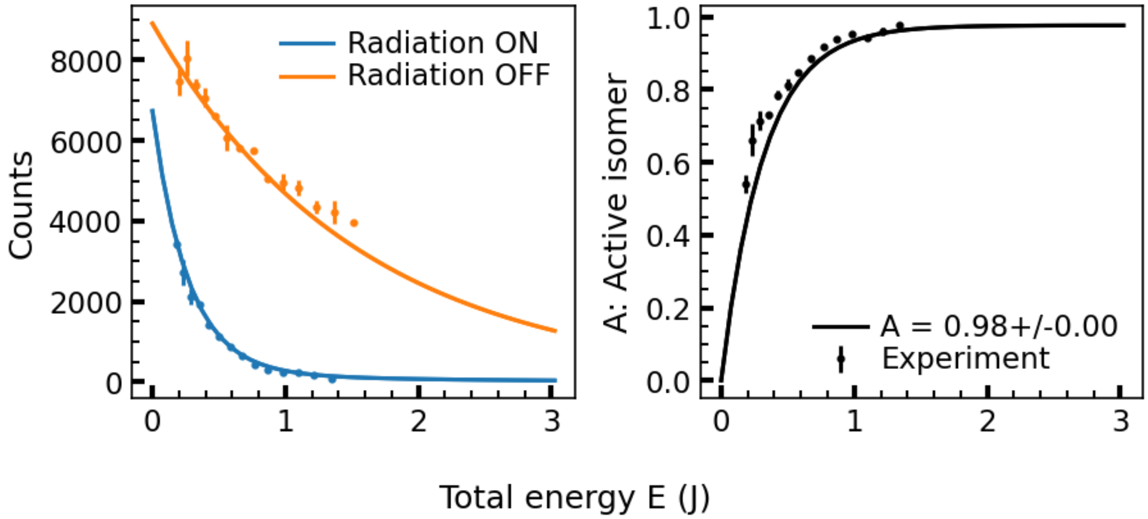

Supplementary Figure 2: Experimental saturation depletion scan of the 920 cm^{-1} mode of benzonitrile$^{\bullet+}$ (*m/z* 103). The on-resonance scan at 920 cm^{-1} (blue) and off-resonance scan at 1010 cm^{-1} (orange) are shown in the left panel. The relative depletion of the active isomer (A) is shown in the right panel as a function of the deposited energy (E). The relative depletion of this band belonging to benzonitrile$^{\bullet+}$ is determined to be 98(±5)%.

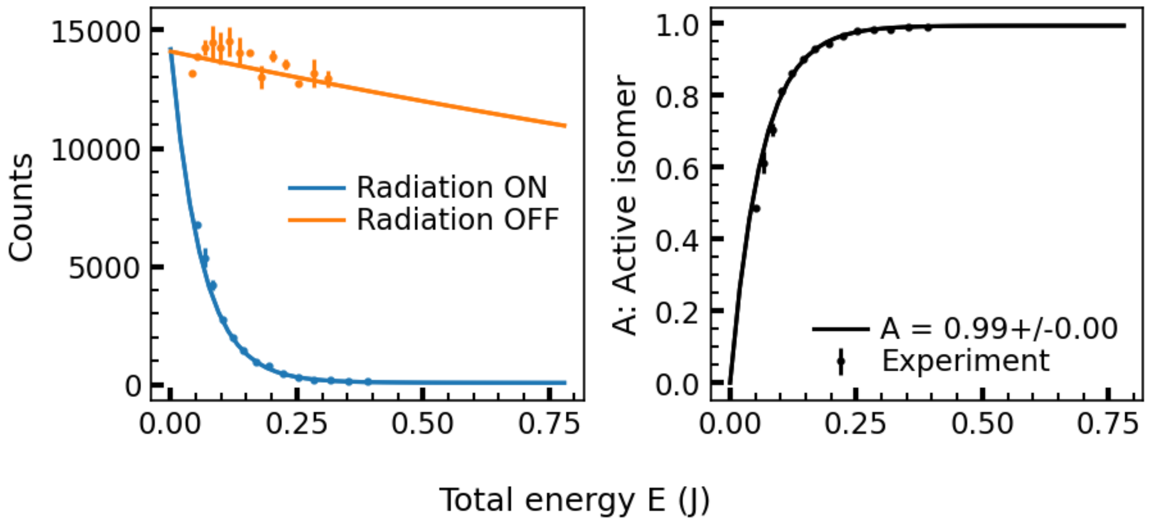

Supplementary Figure 3: Experimental saturation depletion scan of the 1436 cm^{-1} mode of benzonitrile$^{\bullet+}$ (*m/z* 103). The on-resonance scan at 1436 cm^{-1} (blue) and off-resonance scan at 1562 cm^{-1} (orange) are shown in the left panel. The relative depletion of the active isomer (A) is shown in the right panel as a function of the deposited energy (E). The relative depletion of this band belonging to benzonitrile$^{\bullet+}$ is determined to be 99(±5)%.

Supplementary Table 1: Experimental and calculated vibrational frequencies of the benzonitrile radical cation ($C_6H_5CN^{\bullet+}$). The anharmonic calculation is performed at the B3LYP-GD3/N07D level of theory. Only the assigned bands are displayed. Some experimental features are assigned to multiple vibrational modes.

Experimental frequencies	Relative intensity per photon	Anharmonic frequencies	Calculated intensity	Mode	Symmetry
604(1)	5.6	613	41	ν_{19}	B_1
708(1)	1.4	-	-	-	-
768(1)	4.0	-	-	-	-
792(1)	6.8	786	212	ν_{18}	B_1
868(1)	1.4	-	-	-	-
920(1)	4.0	-	-	-	-
957(1)	2.6	955	7	ν_{17}	B_1
968(1)	5.7	969	137	ν_{10}	A_1
986(1)	2.9	978	31	ν_9	A_1
1032(1)	1.4	-	-	-	-
1165(1)	7.2	1177	2	ν_8	A_1
1224(1)	2.0	1215, 1218	1, 3	$\nu_{30} + \nu_{33}$, $\nu_{18} + \nu_{20}$	A_1, A_1
1291(1)	1.0	-	-	-	-
1375(1)	3.5	1384, 1389, 1391	9, 5, 7	ν_{26}, $\nu_{17} + \nu_{20}$, $\nu_{14} + \nu_{19}$	B_2, A_1, B_2
1417(1)	6.8	-	-	-	-
1436(1)	18.3	1435	266	ν_6	A_1
1484(1)	6.5	1490	27	ν_{25}	B_2
1543(1)	0.9	1525	12	ν_5	A_1
1717(1)	5.2	1755	30	$\nu_{28} + \nu_{32}$	A_1
2120(1)	34.6	2171	283	ν_4	A_1

Error of the Gaussian fit in parentheses in the unit of the last digit. Frequencies are shown in wavenumber (cm^{-1}) and the calculated intensity is given in km/mol.

Fitting the kinetic curves with an ODE model

Multiple coupled ordinary differential equations (ODE) are defined that describe the observed reactions as shown below. The ODE model is solved for a pseudo-first-order approximation and the solutions are fitted to the measured data to yield the first-order rate coefficients (s^{-1}). The second-order rate coefficients are determined by plotting the first-order rate against the acetylene number density as shown in **Supplementary Figure 4**. A linear fit of the data yields the second-order rate coefficient of the reaction.

$$\frac{d[C_7H_5N^{\bullet+}]}{dt} = -k_{RA}[C_7H_5N^{\bullet+}][C_2H_2] + k_{CID}[C_9H_7N^{\bullet+}][C_2H_2 + He]$$

$$\frac{d[C_9H_7N^{\bullet+}]}{dt} = +k_{RA}[C_7H_5N^{\bullet+}][C_2H_2] - k_{RA,C11}[C_9H_7N^{\bullet+}][C_2H_2]$$

$$- k_{bi}[C_9H_7N^{\bullet+}][C_2H_2] - k_{CID}[C_9H_7N^{\bullet+}][C_2H_2 + He]$$

$$\frac{d[C_{11}H_8N^{+}]}{dt} = +k_{bi}[C_9H_7N^{\bullet+}][C_2H_2]$$

$$\frac{d[C_{11}H_9N^{\bullet+}]}{dt} = +k_{RA,C11}[C_9H_7N^{\bullet+}][C_2H_2]$$

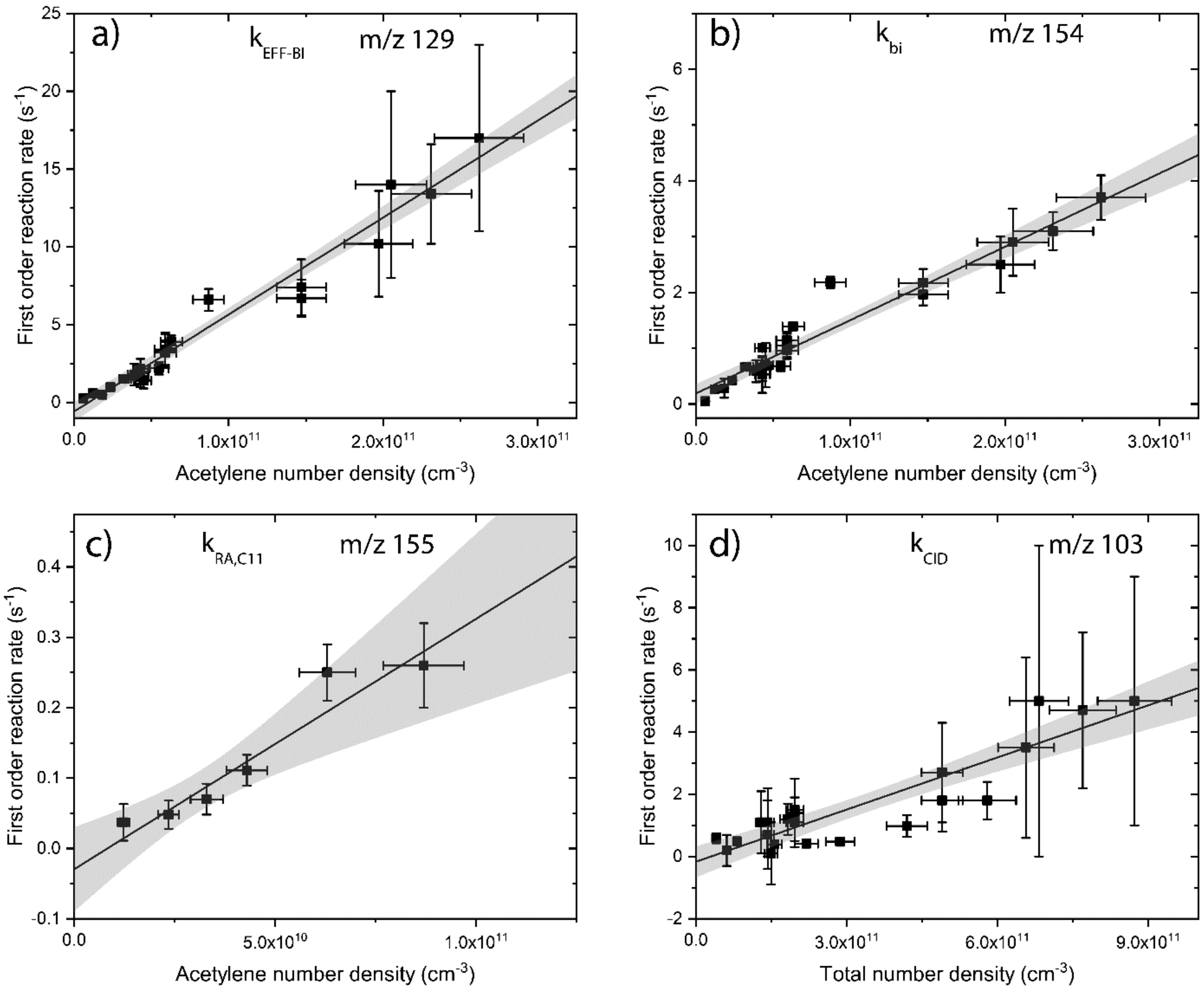

Supplementary Figure 4: Second-order reaction rate coefficients obtained using a linear fit: (a) k_{EFF-BI} to *m/z* 129 (b) k_{bi} to *m/z* 154 (c) $k_{RA,C11}$ to *m/z* 155 and (d) k_{CID} to *m/z* 103. The error bars indicate the 1σ errors and the grey shaded bands define the 2σ uncertainty regions of the fits.

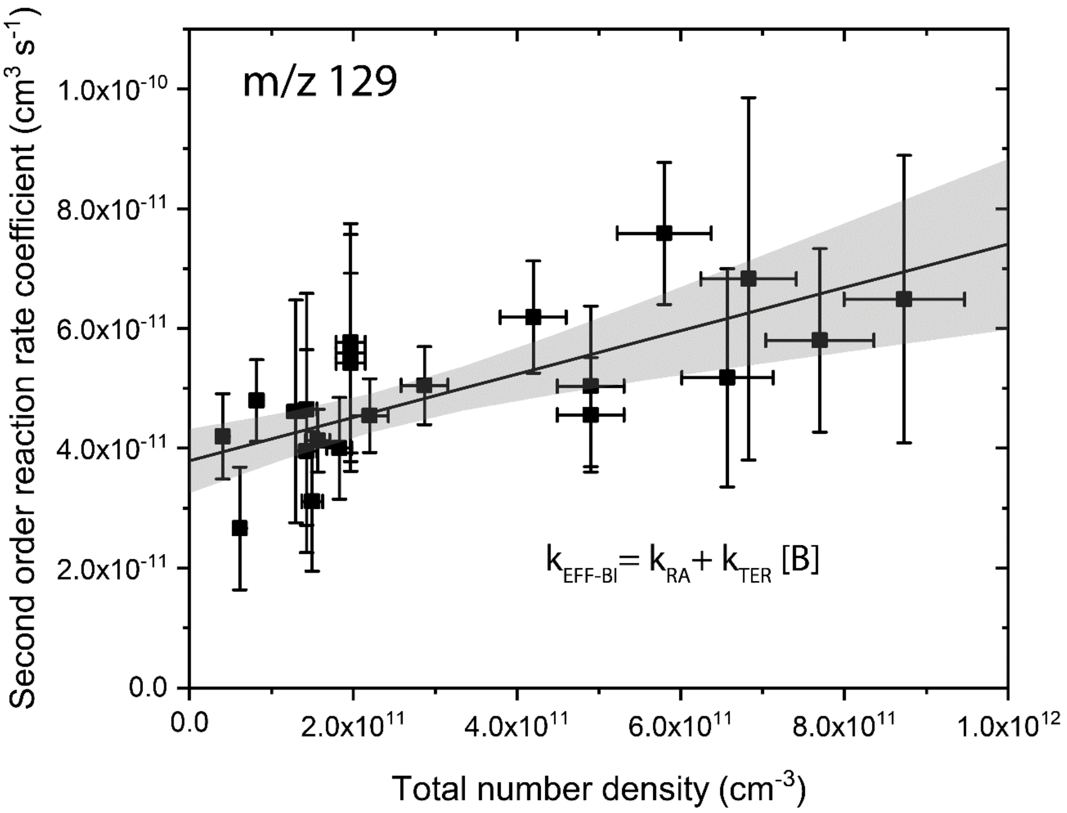

Supplementary Figure 5: Second-order reaction rate coefficient of the reaction to *m/z* 129 plotted against the total number density of acetylene and helium. The linear fit is used to extract the radiative and termolecular stabilization terms of the overall effective bimolecular association reaction rate constant (k_{EFF-BI}) according to the formula displayed in the figure. Assuming no saturation, the effective bimolecular rate constant can be described according to formula $k_{EFF-BI} = k_{RA} + k_{TER}[\mathrm{B}]$ with k_{RA} the radiative association rate constant, k_{TER} the termolecular collisional stabilization and [B] the total number density. Values from the fit are: k_{RA} = 3.8(±0.4) x 10^{-11} $cm^{-3}s^{-1}$ and k_{TER} = 3.6(±1.2) x 10^{-23} $cm^{-6}s^{-1}$. The error bars indicate the 1σ errors and the grey shaded bands define the 2σ uncertainty regions of the fits.

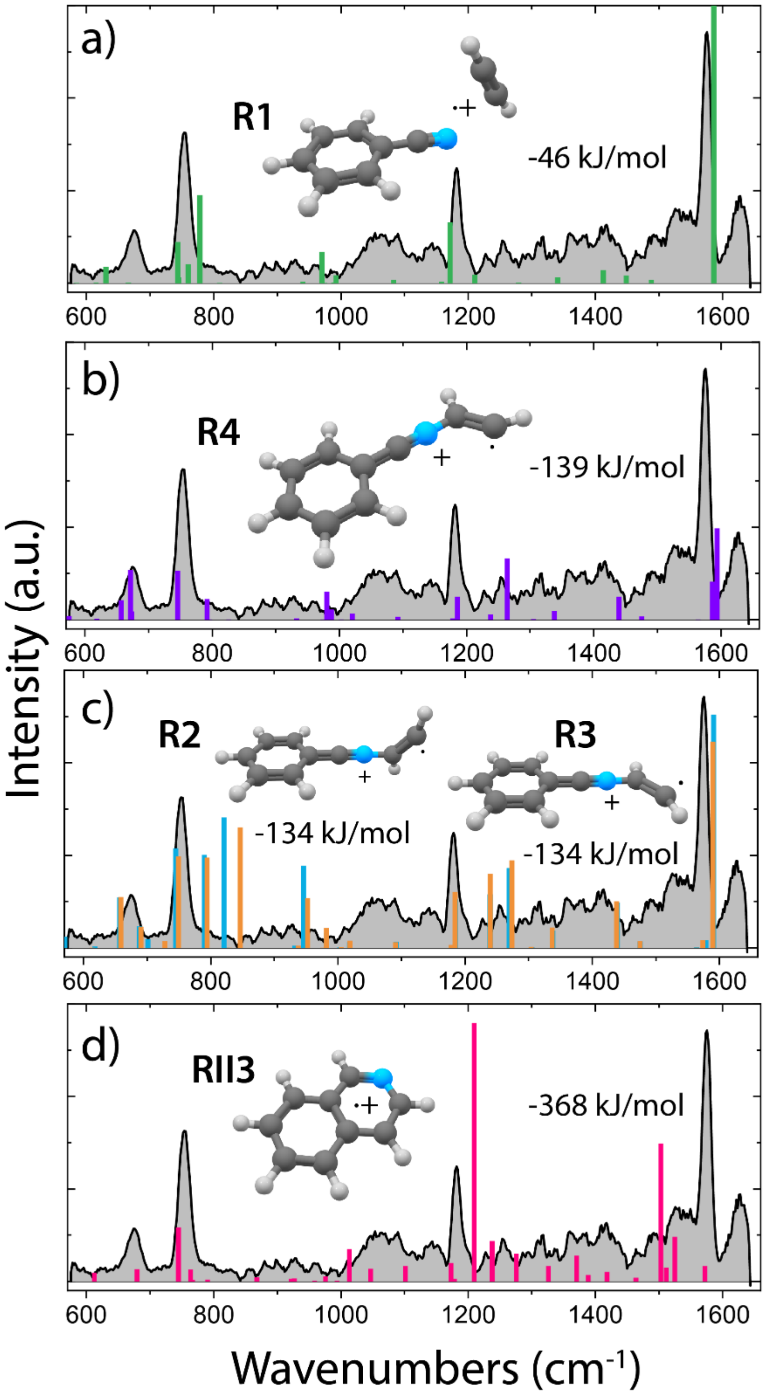

Supplementary Figure 6: Experimental infrared fingerprint spectrum (grey) of the intermediate with *m/z* 129. Calculated vibrational modes are shown for (a) the assigned noncovalent acetylene benzonitrile$^{\bullet+}$ complex (**R1**, green), (b) N-acetylene-benzonitrile$^{\bullet+}$ (**R4**, purple), (c) two N-acetylene-benzonitrile$^{\bullet+}$ conformers (**R2**, **R3**, orange and blue, respectively) and (d) isoquinoline$^{\bullet+}$ (**RII3**, pink). The calculations have been performed at the harmonic B3LYP-GD3/N07D level of theory. The zero-point vibrational energy corrected electronic energies of the molecules are shown relative to the energy of the entrance channel.

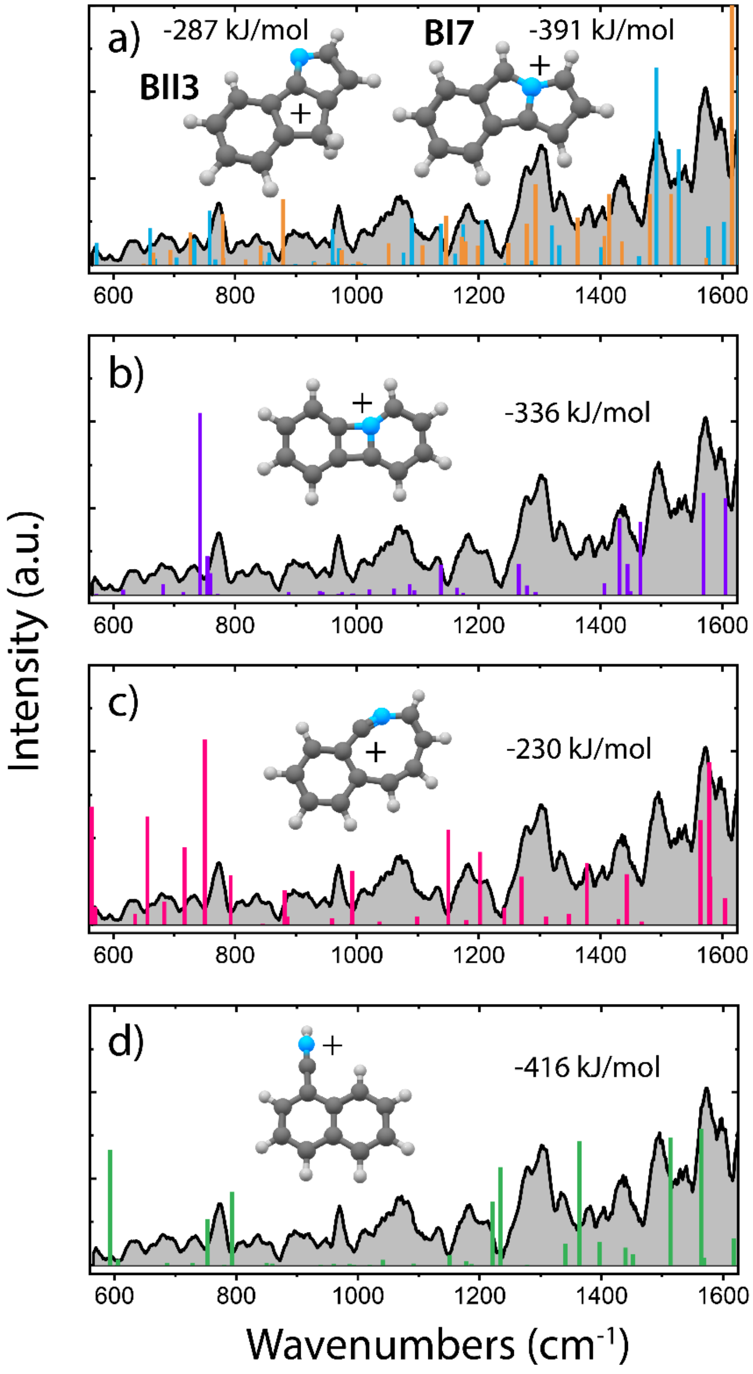

Supplementary Figure 7: Experimental infrared fingerprint spectrum (grey) of the reaction product with *m/z* 154. Calculated vibrational modes are shown for (a) benzo-N-pentalene$^+$ (**BI7**, blue) and benzo-N-pentaleneCH$_2^+$ (**BII3**, orange), (b) fused phenyl-pyridine$^+$ (purple), (c) dehydrogenated 2-benzoazocine$^+$ (pink) and (d) protonated 1-cyano-naphthalene$^+$ (green). The calculations have been performed at the harmonic B3LYP-GD3/N07D level of theory. The zero-point vibrational energy corrected electronic energies of the molecules are shown relative to the energy of the entrance channel.

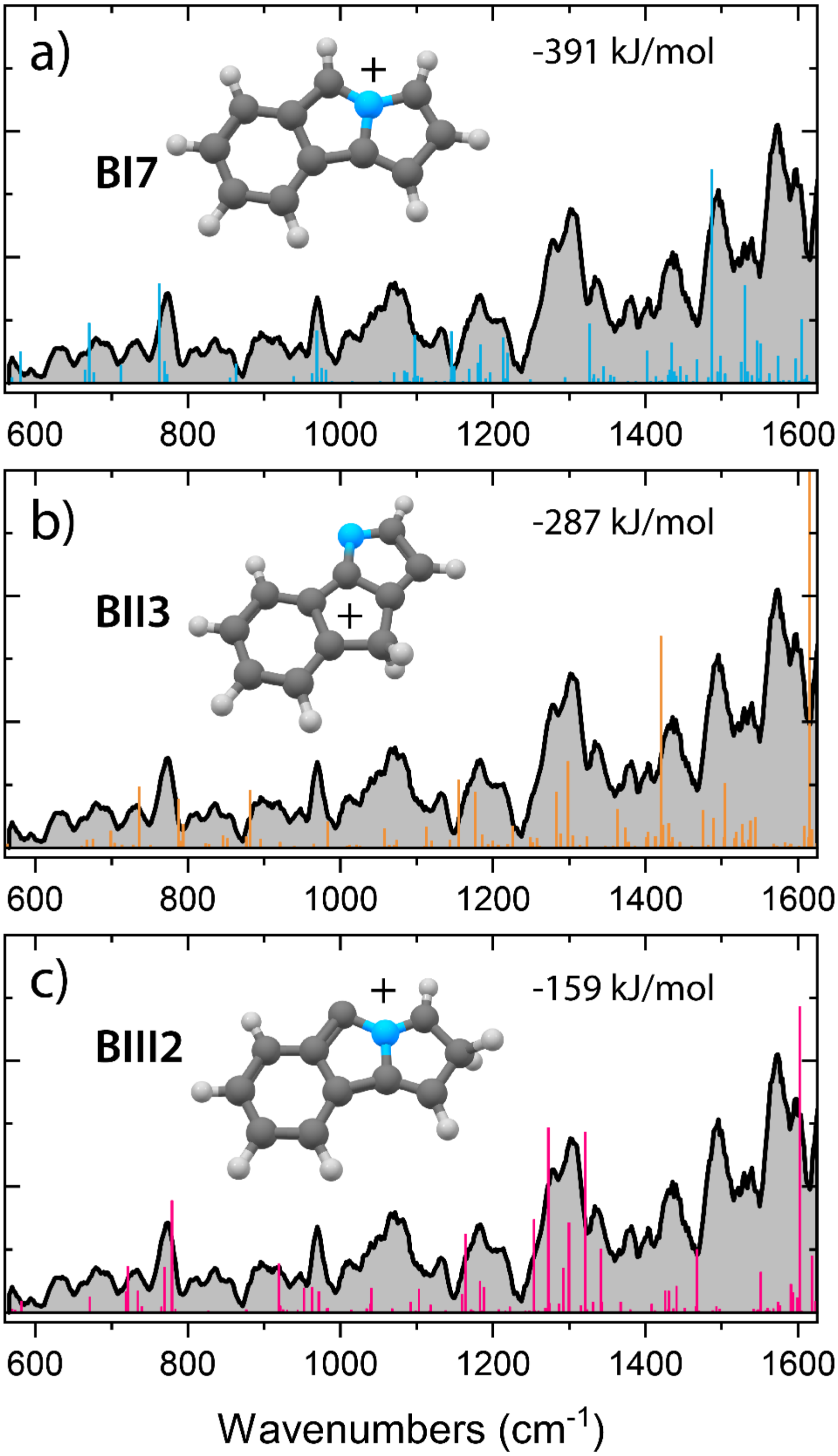

Supplementary Figure 8: Experimental infrared fingerprint spectrum (grey) of the reaction product with *m/z* 154. Calculated anharmonic vibrational modes are shown for (a) benzo-N-pentalene$^+$ (**BI7**, blue), (b) benzo-N-pentaleneCH_2^+ (**BII3**, orange) and (c) a different benzo-N-pentaleneCH_2^+ isomer (**BIII2**, pink). The calculations have been performed at the anharmonic B3LYP-GD3/N07D level of theory. The zero-point vibrational energy corrected electronic energies of the molecules are shown relative to the energy of the entrance channel.

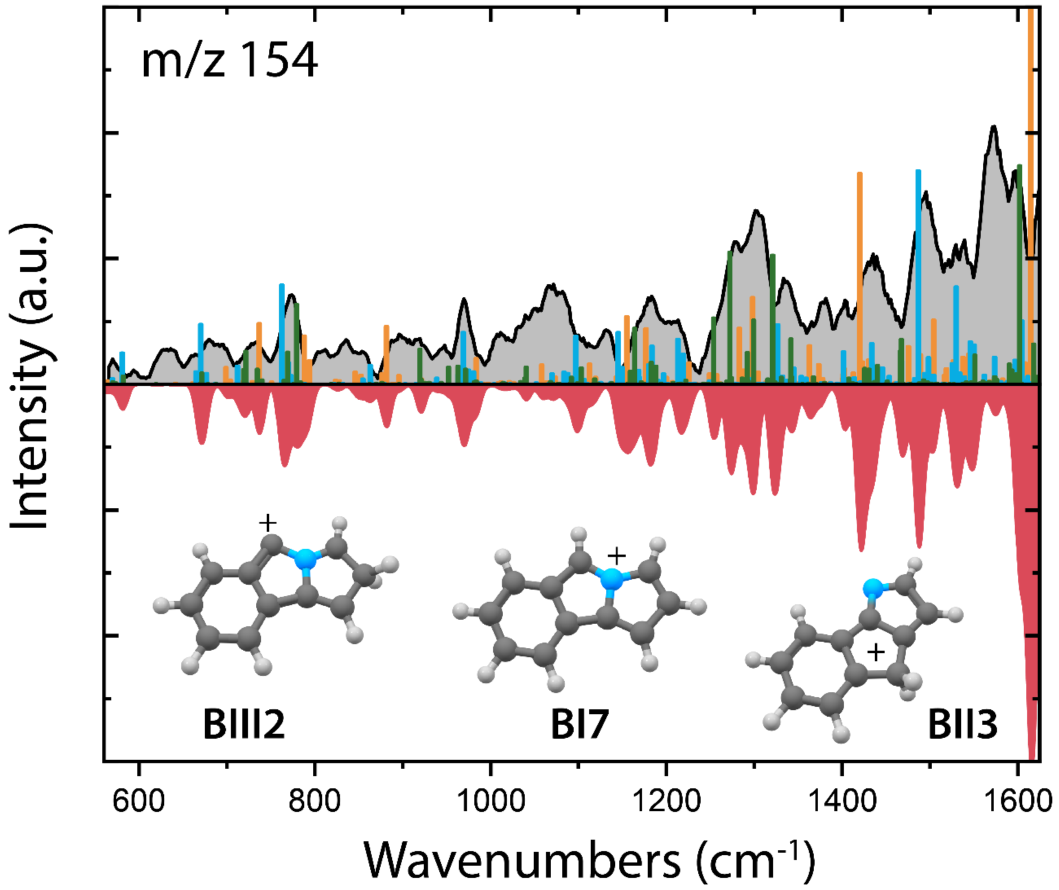

Supplementary Figure 9: Experimental infrared fingerprint spectrum (grey) of the reaction product with *m/z* 154. Calculated anharmonic vibrational modes are shown for the benzo-N-pentalene$^+$ (**BI7**, blue), benzo-N-pentaleneCH_2^+ (**BII3**, orange) and benzo-N-pentaleneCH_2^+ isomer (**BIII2**, green). The calculations have been performed at the anharmonic B3LYP-GD3/N07D level of theory. A convoluted spectrum with equal contributions of all three isomers is shown in red.

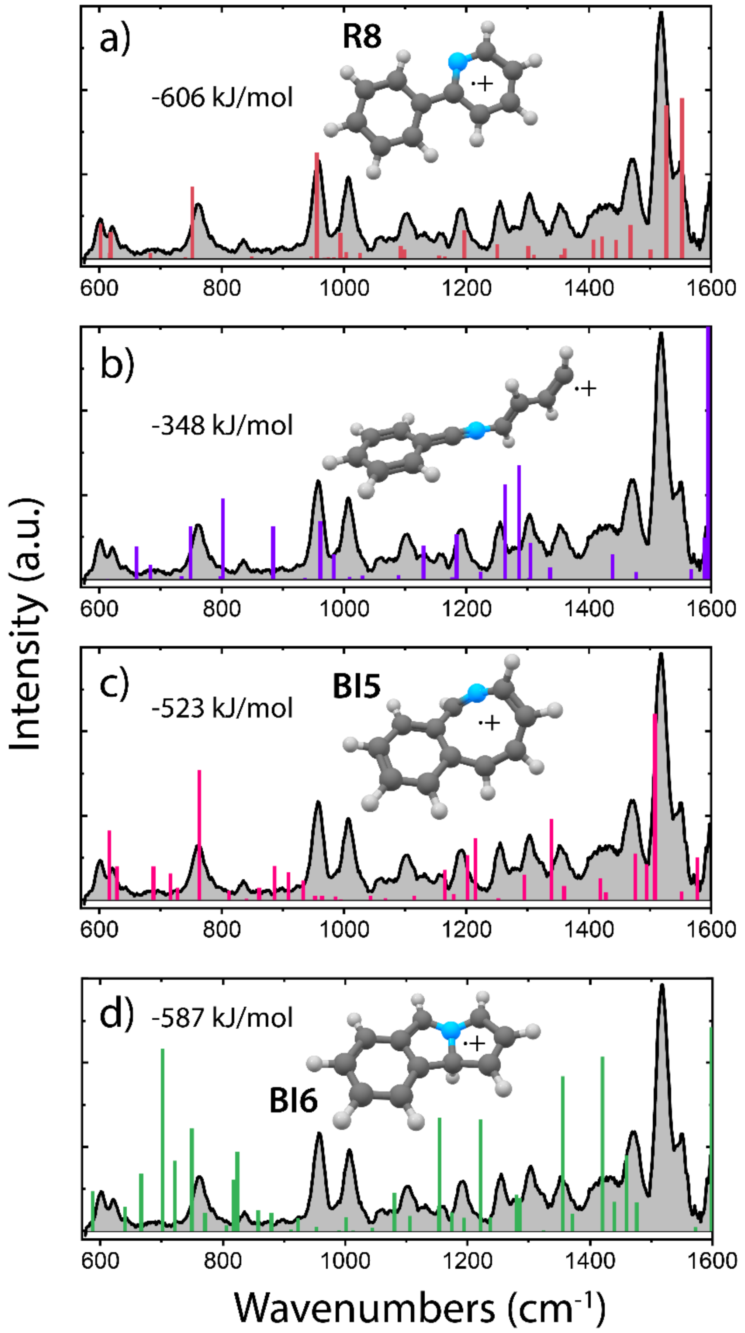

Supplementary Figure 10: Experimental infrared fingerprint spectrum (grey) of the reaction product with *m/z* 155. Calculated vibrational modes are shown for (a) the assigned 2-phenylpyridine$^{\bullet+}$ (**R8**, red), (b) N-C_4H_4-benzonitrile$^{\bullet+}$ (purple), (c) 2-benzoazocine$^{\bullet+}$ (**BI5**, pink) and (d) protonated benzo-N-pentalene$^{\bullet+}$ (**BI6**, green). The calculations have been performed at the harmonic B3LYP-GD3/N07D level of theory. The zero-point vibrational energy corrected electronic energies of the molecules are shown relative to the energy of the entrance channel.

Supplementary Table 2: Zero-point vibrational energy corrected electronic energies of the minima and transition states shown in **Figure 4**. Calculated at the B3LYP-GD3/N07D level of theory.

Minimum	Energy (kJ/mol)
**R1**	-46
**TSR1**	-45
**R2**	-134
**R3**	-134
**TSR2**	-128
**R4**	-139
**R5**	-160
**TSR3**	-155
**R6**	-332
**TSR4**	-328
**R7**	-346
**TSR5**	-339
**R8**	-606

Supplementary Table 3: Zero-point vibrational energy corrected electronic energies of the minima and transition states shown in **Figure 5**. Calculated at the B3LYP-GD3/N07D level of theory.

Minimum	Energy (kJ/mol)	Minimum	Energy (kJ/mol)	Minimum	Energy (kJ/mol)
**R7**	-346				
**TSBI1**	-269				
**BI1**	-374				
**TSBI2**	-279				
**BI2**	-470				
**TSBI3**	-338	**TSBII1**	-406		
**BI3**	-434	**BII1**	-529		
**TSBI4**	-309	**TSBII2**	-405		
**BI4**	-438	**BII2**	-453		
**TSBI5**	-306	**TSBII3**	-285	**TSBIII1**	-214
**BI5**	-523	**BII3**	-287	**BIII1**	-278
**TSBI6**	-398			**TSBIII2**	-136
**BI6**	-587			**BIII2**	-159
**TSBI7**	-380				
**BI7**	-391				

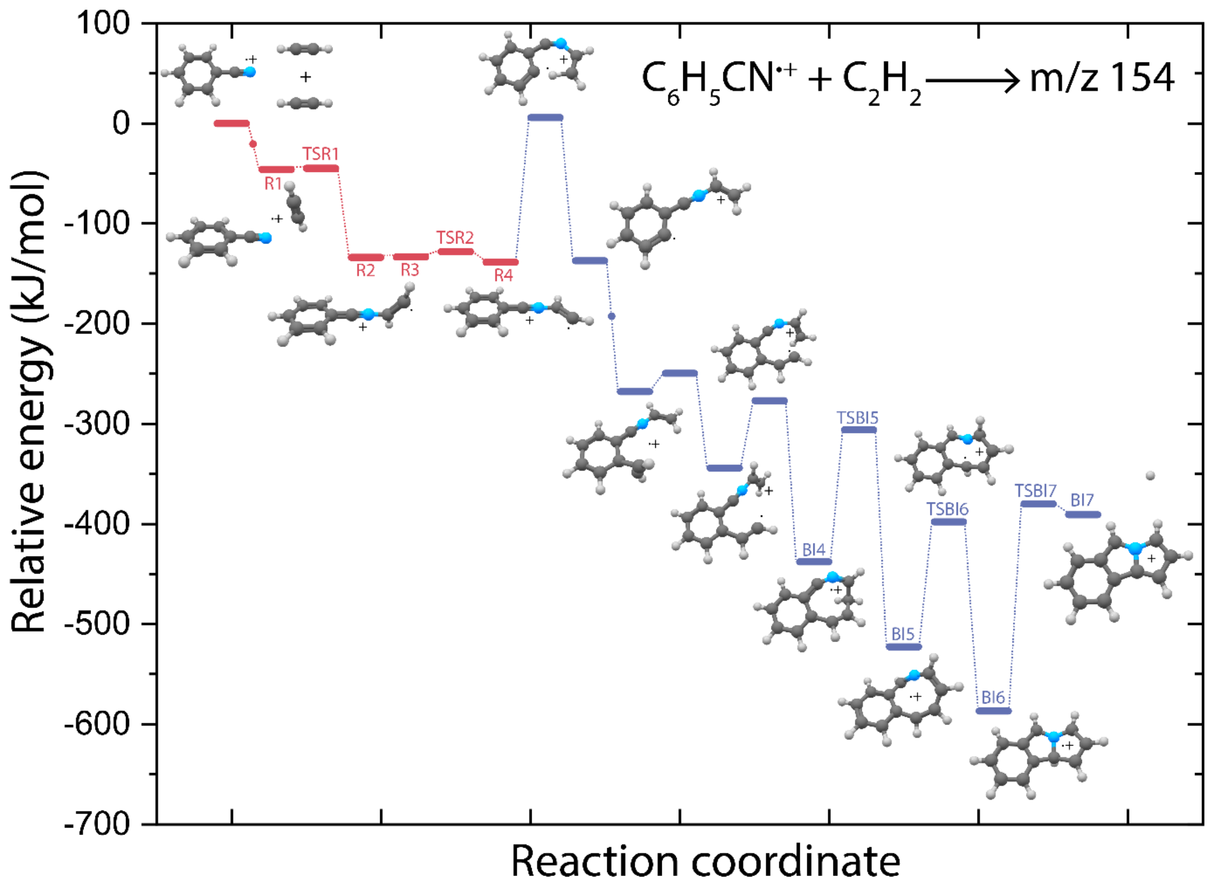

Supplementary Figure 11: Alternative PES of the benzonitrile$^{\bullet+}$ ($C_6H_5CN^{\bullet+}$) with acetylene (C_2H_2) reaction towards benzo-N-pentalene$^+$ (**BI7**, *m/z* 154, purple pathway). The first part of the reaction pathway (red) follows the first part of the calculated path from **Figure 4**. The small dots along the pathway display the addition of a new acetylene molecule.

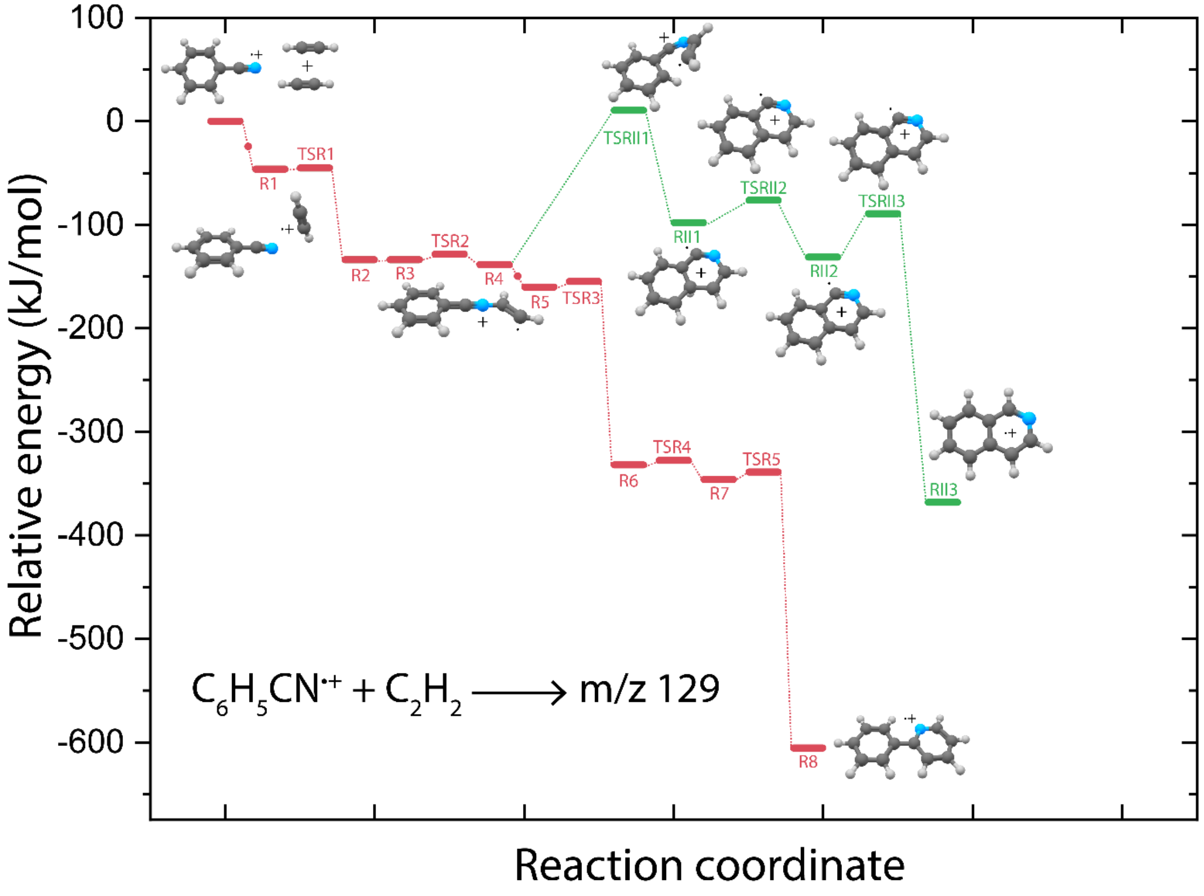

Supplementary Figure 12: Potential energy surface of benzonitrile$^{\bullet+}$ ($C_6H_5CN^{\bullet+}$) with acetylene (C_2H_2) reaction towards the alternative product isoquinoline$^{\bullet+}$ (**RII3**, *m/z* 129, green pathway). The first part of the reaction pathway (red) follows what was calculated in **Figure 4**. The small dots along the pathway display the addition of a new acetylene molecule.

References

(1) Marimuthu, A. N.; Sundelin, D.; Thorwirth, S.; Redlich, B.; Geppert, W. D.; Brünken, S. Laboratory Gas-Phase Vibrational Spectra of [C3H3]+ Isomers and Isotopologues by IRPD Spectroscopy. *J. Mol. Spectrosc.* **2020**, *374*, 111377. https://doi.org/10.1016/j.jms.2020.111377.
